# A tick saliva serpin, *Ixs*S17 inhibits host innate immune system proteases and enhances host colonization by Lyme disease agent

**DOI:** 10.1371/journal.ppat.1012032

**Published:** 2024-02-23

**Authors:** Thu-Thuy Nguyen, Tae Heung Kim, Emily Bencosme-Cuevas, Jacquie Berry, Alex Samuel Kiarie Gaithuma, Moiz Ashraf Ansari, Tae Kwon Kim, Lucas Tirloni, Zeljko Radulovic, James J. Moresco, John R. Yates, Albert Mulenga

**Affiliations:** 1 Department of Veterinary Pathobiology, School of Veterinary Medicine and Biomedical Sciences, Texas A&M University, College Station, Texas, United States of America; 2 Department of Diagnostic Medicine/Pathobiology, College of Veterinary Medicine, Kansas State University, Manhattan, Kansas, United States of America; 3 Tick-Pathogen Transmission Unit, Laboratory of Bacteriology, NIAID, Hamilton, Montana, United States of America; 4 Department of Biology, Stephen F. Austin State University, Nacogdoches, Texas, United States of America; 5 Center for Genetics of Host Defense, UT Southwestern Medical Center, Dallas, Texas, United States of America; 6 Department of Molecular Medicine, The Scripps Research Institute, La Jolla, California, United States of America; University of North Dakota School of Medicine and Health Sciences, UNITED STATES

## Abstract

Lyme disease (LD) caused by *Borrelia burgdorferi* is among the most important human vector borne diseases for which there is no effective prevention method. Identification of tick saliva transmission factors of the LD agent is needed before the highly advocated tick antigen-based vaccine could be developed. We previously reported the highly conserved *Ixodes scapularis* (*Ixs*) tick saliva serpin (S) 17 (*Ixs*S17) was highly secreted by *B*. *burgdorferi* infected nymphs. Here, we show that *Ixs*S17 promote tick feeding and enhances *B*. *burgdorferi* colonization of the host. We show that *Ixs*S17 is not part of a redundant system, and its functional domain reactive center loop (RCL) is 100% conserved in all tick species. Yeast expressed recombinant (r) *Ixs*S17 inhibits effector proteases of inflammation, blood clotting, and complement innate immune systems. Interestingly, differential precipitation analysis revealed novel functional insights that *Ixs*S17 interacts with both effector proteases and regulatory protease inhibitors. For instance, r*Ixs*S17 interacted with blood clotting proteases, fXII, fX, fXII, plasmin, and plasma kallikrein alongside blood clotting regulatory serpins (antithrombin III and heparin cofactor II). Similarly, r*Ixs*S17 interacted with both complement system serine proteases, C1s, C2, and factor I and the regulatory serpin, plasma protease C1 inhibitor. Consistently, we validated that r*Ixs*S17 dose dependently blocked deposition of the complement membrane attack complex via the lectin complement pathway and protected complement sensitive *B*. *burgdorferi* from complement-mediated killing. Likewise, co-inoculating C3H/HeN mice with r*Ixs*S17 and *B*. *burgdorferi* significantly enhanced colonization of mouse heart and skin organs in a reverse dose dependent manner. Taken together, our data suggests an important role for *Ixs*S17 in tick feeding and *B*. *burgdorferi* colonization of the host.

## Author summary

Ticks feed on animals and humans for their survival. During blood meal feeding, ticks inject saliva along with disease causative agents into the hosts. Here, we demonstrate that *I*. *scapularis* tick saliva protein, *Ixs*S17 inhibits host innate immune system proteases and enhances *B*. *burgdorferi* colonization of the host. Recombinant *Ixs*S17 (r*Ixs*S17) inhibits blood clotting and inflammation systems serine proteases including pancreatic trypsin and trypsin IV (~100%), blood clotting factor Xa and XIa (~60–80%), plasmin and cathepsin G (~50%). Similarly, r*Ixs*S17 interacts with complement system factors, C1s, C2 and factor I and blocks complement membrane attack complex via the lectin complement pathway by up to 97%. We found that, in the mouse model for Lyme disease, r*Ixs*S17 significantly increases *B*. *burgdorferi* colonization of mouse heart and ear tissues by 5.7 and 2.3 times. Taken together, we conclude that *Ixs*S17 is a key protein in tick feeding and *B*. *burgdorferi* colonization of the host, and thus, a potential target antigen for developing tick antigen-based vaccines against Lyme disease agent transmission.

## Introduction

Ticks and tick-borne diseases (TBD) impact public and veterinary health globally. Among those, Lyme disease (LD) caused by *Borrelia* species is one of the most important human TBD that has the most world-wide public health impact. The spirochete, *Borrelia burgdorferi* that is transmitted by *Ixodes* spp. ticks is responsible for LD in United States (US) and Europe, while *B*. *afzelii* and *B*. *garinii* are responsible for LD in Eurasia [1–3]. Recently, a second LD pathogen *B*. *mayonii* was described in the US [4]. Like other TBD agents, except a vaccine against tick-borne encephalitis approved by FDA in United States in 2021, there is currently no effective human vaccine against the LD agent.

In the absence of effective vaccines against the LD agent, avoidance of infectious tick bites is the only prevention method against LD currently. Despite a plethora of methods aimed at reducing infectious tick bites [5–7], LD cases have continued to increase. Confirmed and probable LD cases reported to the US Centers for Disease Control and Prevention have steadily risen from just under 20,000 in 1996 to more than 40,000 annual cases since 2008 (www.cdc.gov). According to insurance database, between 2010–2018, 476,000 LD cases were diagnosed and treated each year, with economic losses estimated at ~$786 million annually [8,9].

Given the ongoing rise in LD cases and search for better preventative measures, tick-antigen based vaccines have emerged among the most promising LD prevention approaches. This is based on evidence that repeatedly infested model animals that acquire immunity against tick feeding are protected against transmission of TBD agents including *B*. *burgdorferi* [10–13]. Similarly, in a recent study, repeatedly infested primates were also protected against *B*. *burgdorferi* transmission [14]. Likewise, active immunization of mice with tick saliva proteins conferred immunity that reduced transmission of LD agents [15–17]. Similarly, tick saliva and tick salivary gland extracts promoted LD agent replication [18,19] and innate immunity evasion *ex vivo* [20], and enhanced organ colonization in needle inoculated mice [21,22]. From these perspectives, tick saliva factors that promote feeding and transmission of TBD agents have been highly sought after [23–27].

To date, there is no evidence of transovarial transmission (or passed from female ticks to larval ticks) of LD agents. Larval ticks acquire the spirochetes during feeding on infected reservoir hosts and then transtadially transmit to nymphs, which in turn transtadially transmit to adult ticks [28]. Major transmission events of *B*. *burgdorferi* occur after the tick has fed for more than 48 h [29–31]. The small size of the nymph tick and pain suppressants in its saliva that mask its presence on human skin allows the tick to go unnoticed and feed long enough for more than 36–48 h to transmit the LD agent [32]. For that reason, although both nymph and adult ticks are capable of transmitting LD agents to the human host, most reported LD cases were associated with infectious nymph bites [3,33]. On this basis, we recently identified tick saliva proteins of *B*. *burgdorferi* infected *I*. *scapularis* nymphs that were secreted every 12 h throughout feeding [25].

This study was initiated to understand functional roles of *I*. *scapularis* tick saliva serine protease inhibitor (Serpin; GenBank accession# EEC18973.1 or XP_002415308.5) in tick feeding and *B*. *burgdorferi* colonization of the host. We later found that *Ixs*S17 was among homologs (orthologs) to *Amblyomma americanum* serpin (AAS) 19 that were characterized by the functional domain reactive center loop (RCL) being 100% conserved in all tick species according to currently available data [34,35]. We also reported that *Ixs*S17 and its homologs are among the proteins being injected into animals by adult *I*. *scapularis* [23], *A*. *americanum* [35], and *Rhipicephalus microplus* [27] ticks. In our recent study, we found that *B*. *burgdorferi* infected *I*. *scapularis* nymphs predominantly secreted *Ixs*S17 at 48h feeding time point when major *B*. *burgdorferi* transmission events are expected [25]. Additionally, we showed that RNAi silencing of *Ixs*S17 [36] and its *A*. *americanum* homolog, AAS19 [23] caused mortality and reduced tick feeding efficiency. This evidence suggested that functions of *Ixs*S17 and its homologs are related to tick feeding and transmission of tick-borne pathogens including *B*. *burgdorferi*. Consistent with functional analyses of *Ixs*S17 homologs in *A*. *americanum* (AAS19; [35]), *R*. *microplus* (RmS-15; [37,38]), and recently in *R*. *haemaphysaloides* (RHS8; [39]) and *I*. *ricinus* (Iripin-8;), we provide new information that *Ixs*S17 is an anticoagulant that is potentiated by binding heparin. Significantly, we further show that *Ixs*S17 promotes *B*. *burgdorferi* colonization of the host by inhibiting host inflammation, blood clotting, and complement system effector proteases.

## Results

### *I*. *scapularis* serpin (*Ixs*S) 17 is not redundant and is conserved across Ixodidae tick species

**Fig 1:**
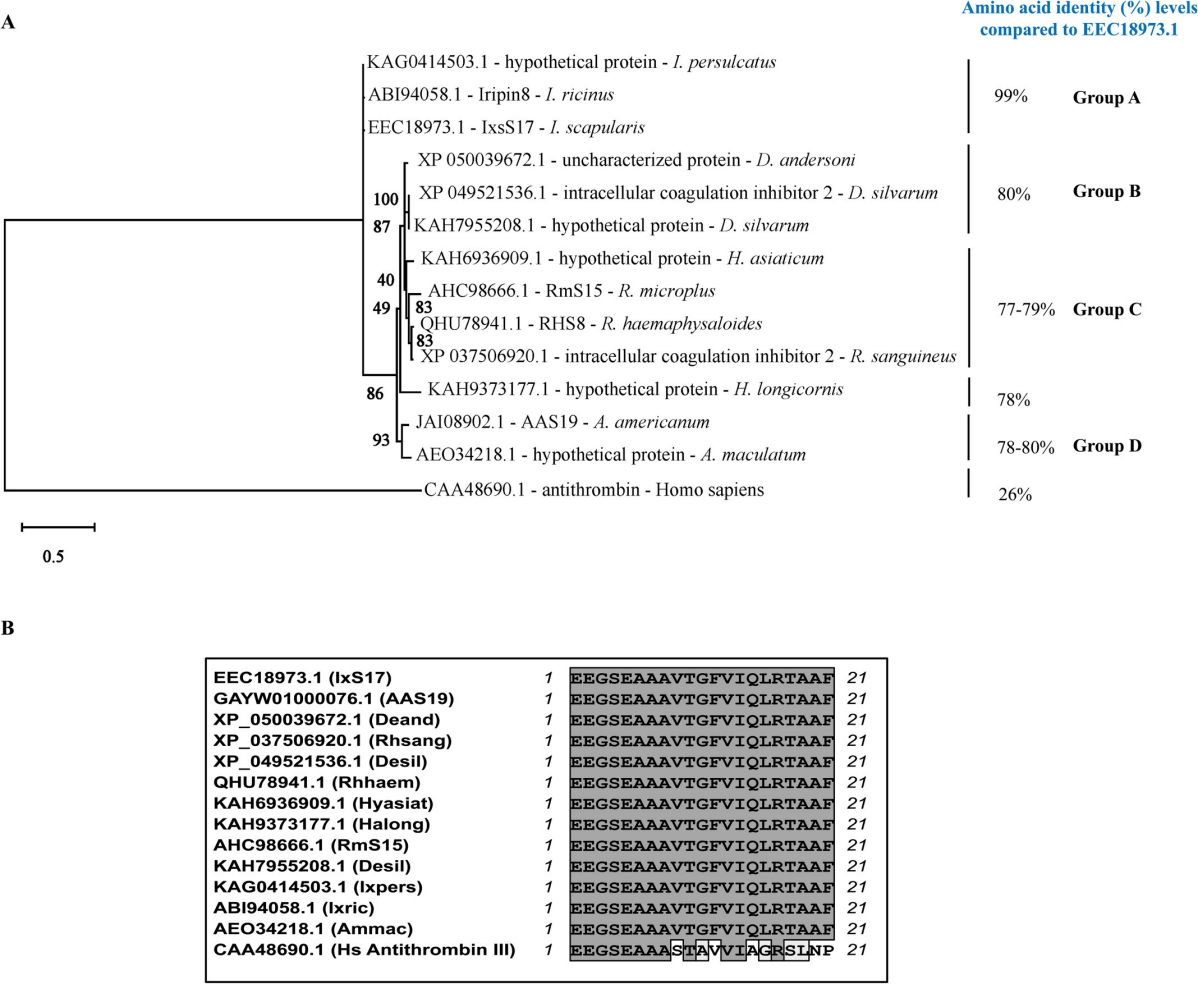
Amino acid sequence analysis of *Ixs*S17. **(**A) Phylogenetic tree was constructed using the MEGA-X software, Maximum Likelihood method and Le_Gascuel_2008 model with Bootstrap set to 1,000 replications. Group A, B, C and D represent amino acid identity levels of *Ixs*S17 to its homologs in percentage. (B) Multiple sequence alignment of *Ixs*S17 reactive center loop (EEGSEAAAVTGFVIQLRTAAF) and its homologs as well as the antithrombin III outlier was done in MacVector using T-Coffee specifications. Amino acids in the grey box are identical.

BLASTP search of *Ixs*S17 (EEC18973.1 or XP_002415308.5) amino acid sequence against entries in GenBank retrieved one sequence match of more than 77% amino acid identity per tick species except for *Dermacentor silvarum*, which has two matches (S1 Fig). The next highest matches to *Ixs*S17 in *I*. *scapularis* and other tick species showed amino acid identity levels of less than 50%. This indicates that *Ixs*S17 and its homologs, in other tick species, are not redundant except for *D*. *silvarum*, which has two matches that differ by an 11 amino acid deletion to *Ixs*S17: KAH7955208.1 and XP_049521536.1 (S1 Fig). With *Homo sapiens* antithrombin III (CAA48690.1) set as an outlier, neighbor joining phylogeny tree segregated *Ixs*S17 in group A with other *Ixodes* spp. tick serpins: *I*. *ricinus* (ABI94058.1) and *I*. *persulcatus* (KAG0414503.1) that show 99% amino acid identity to *Ixs*S17 (Fig 1A, group A). *Ixs*S17 is 80% identical to its homologs in metastriata ticks including *D*. *andersoni* (XP_050039672.1) and *D*. *silvarum*, (KAH7955208.1 and XP_049521536.1) in group B. Likewise, *Ixs*S17 is 77–79% identical to *Hyalomma asiaticum* (KAH6936909.1), *Rhipicephalus microplus* serpin 15 (RmS15; AHC98666.1), *R*. *sanguineous* (XP_037506920.1), and *R*. *haemaphysoloides* (QHU78941.) in cluster C (Fig 1A). Finally, in group D, *Ixs*S17 is 78–80% identical to *Amblyomma americanum* (AAS19; GAYW01000076.1), *A*. *maculatum* (AEO34218.1), *A*. *triste* (A0A023GPF9), *A*. *cajeenense* (A0A023FM57), and *Haemaphysalis longicornis* (KAH9373177.1) (Fig 1A, group D). Although overall amino acid identity is below 100% (S1 Fig), the 21 amino acid sequence of *Ixs*S17 functional reactive center loop (RCL: EEGSEAAAVTGFVIQLRTAAF) is 100% conserved in all tick serpins analyzed in this study (Fig 1B). *Ixs*S17 was initially described among 45 *I*. *scapularis* serpin sequences that were extracted genome contigs [40]. In this manuscript, we show that the *I*. *scapularis* genome (RefSeq GCF-016920785.2) encode for 62 serpins including *Ixs*S17 as revealed by unique serpin RCLs (S1 Table). Pairwise and global alignment of the 62 RCLs with coverage set to between 80–100% confirmed that *Ixs*S17 RCL was not redundant as all other RCLs are 29–52% identical to *Ixs*S17.

When compared to its homologs in other tick species both EEC18973.1 [41, 42] and XP_002415308.5 [43] have a 53 amino acid sequence extension at the amino terminus end. SignalP 6.0 software did not identify the signal peptide in EEC18973.1 or XP_002415308.5 unless the first 53 amino acids were removed (S2A Fig). However, the subcellular localization prediction software DeepLoc-2.0 indicated that EEC18973.1 or XP_002415308.5 were an extra cellular protein with signal peptides at the position 50 to 70 (S2B Fig). In this study we characterized mature EEC18973.1 sequence with the first 70 amino acid sequences removed.

### Yeast expressed r*Ix*sS17 inhibits trypsin-like proteases and its inhibitory function is affected by hexa-histidine tag location

**Fig 2:**
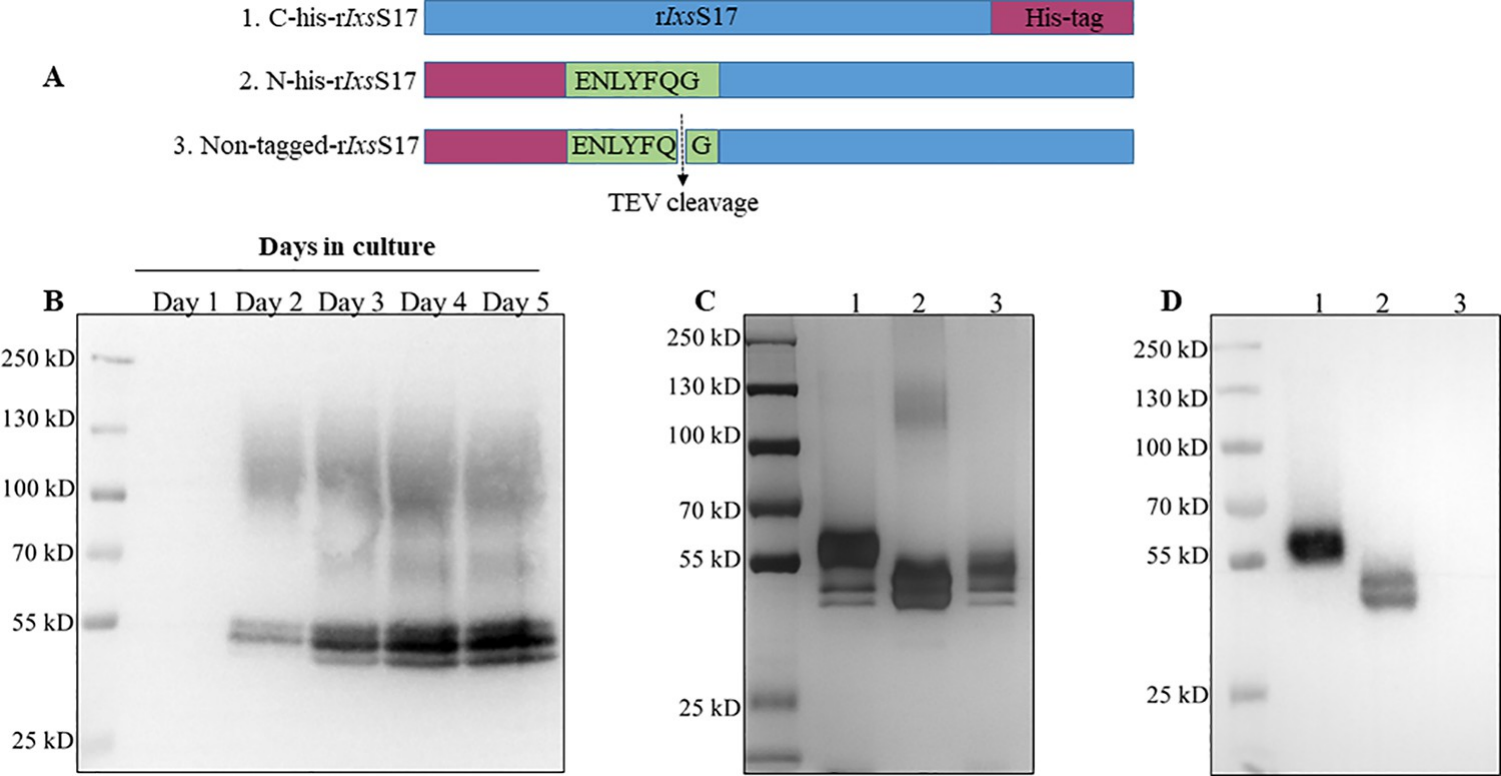
Expression and affinity purification of recombinant (r) *Ixs*S17. (A) Graphical illustration of three different r*Ixs*S17 expression constructs that were custom synthesized: (1) C-terminal hexa-histidine tag, (2) N-terminal hexa-histidine tag and Tobacco Etch Virus (TEV) cutting site (ENLYFQG) included, and (3) hexa-histidine tag is cleaved off at the TEV cutting site in the non-tagged r*Ixs*S17. Please note all three recombinant constructs contain full-length sequence of r*Ixs*S17. (B) Western blotting analysis of daily expression of r*Ixs*S17 in *Pichia pastoris* culture. Culture (1 mL) were precipitated by ammonium sulfate saturation and resolved on 10% SDS-PAGE. r*Ixs*S17 were detected in western blot using the HRP-conjugated monoclonal antibody to the hexa-histidine tag. (C) Silver staining and (D) Western blotting analysis of affinity purified r*Ixs*S17. The hexa-histidine tag was detected in the C-terminal (Lane 1) and N-terminal-His-r*Ixs*S17 (Lane 2) but not in the non-tagged r*Ixs*S17 (Lane 3).

Canonical mode of serpin inhibitory activity is mechanical disruption of the target protease which starts with the C-terminal reaction center loop (RCL) irreversibly trapping the target protease [44]. Determined to investigate the effect of the hexa-histidine (His) fusion tag on inhibitory functions of r*Ixs*S17, we successfully expressed, and affinity purified three r*Ixs*S17 constructs: (1) the hexa-histidine tag located at the N- terminal or (2) C-terminal ends or (3) cleaved off using the inhouse produced Tobacco etch virus (TEV) protease (Fig 2). The r*Ixs*S17 are glycosylated like other tick (*Ixs*S-1E1 [AID54718.1], AAS19 [JAI08902.1], AAS27 [GAGD01011247.1], and AAS41 [JAI08957.1]) and human serpins (antithrombin III and vaspin) [24,35,45–48]. After deglycosylation treatment, protein sizes reduced ~ 2.5–5.0 kDa (S3 Fig). Glycosylation is the most common post-translational modification of proteins when the carbohydrate units are attached to the protein backbone either by N- or O-glycosidic bonds or both [49,50]. In serpins, glycosylation is important for proper protein secretion, stability, and their half-life extension [46,51].

**Fig 3:**
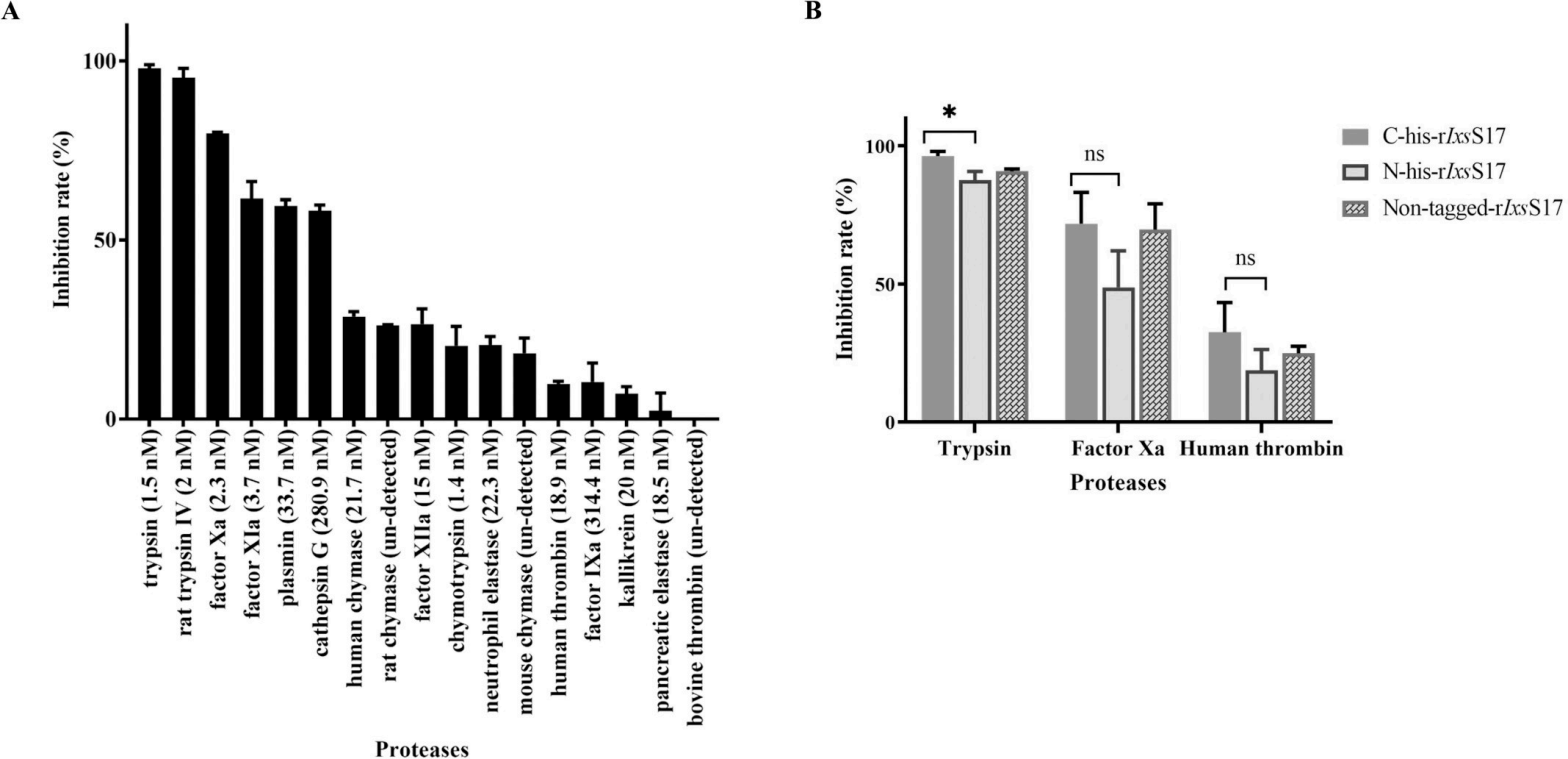
Inhibition profiling of r*Ixs*S17 against 17 serine proteases related to host responses during tick feeding. (A) Inhibition rates of C-terminal-Histidine tagged r*Ixs*S17 (1 μM) against 17 serine proteases (with indicated concentrations) in the substrate hydrolysis assays. Substrate hydrolysis was monitored at *A*_405nm_ every 11s for 30 min at 30°C. Inhibition rate was calculated using the formula: 100-V_i_/V_0_ x 100, where V_i_ = activity in the present of, and V_0_ in the absence of r*Ixs*S17. (B) Inhibition activity of C-terminal, N-terminal and non-Histidine tagged r*Ixs*S17 against trypsin, factor Xa and human thrombin was determined using the substrate hydrolysis assay. Data represents mean ± SEM calculated from 3 biological replicates. The difference was analyzed using ANOVA in GraphPad Prism 9 and is statistically significant when P value ≤ 0.05.

We initially used the C-terminal hexa-His-tagged r*Ixs*S17 in substrate hydrolysis assays of 17 serine proteases related to host responses against tick feeding (Fig 3A and S2 Table). This screen showed that r*Ixs*S17 (1 μM) inhibited pancreatic trypsin (1.5 nM) by 96–100%, rat skin trypsin IV (2.0 nM; in house expressed) by 90–99%, blood clotting factor (f) Xa (2.3 nM) by 79–80% followed by inhibition of blood clotting fXIa (3.7 nM), plasmin (33.7 nM), and cathepsin G (281 nM) by 52–65%, 55–61%, 56–61% respectively. Next, r*Ixs*S17 also inhibited human chymase (21 nM) by 26–31%, as well as native purified rat and mouse chymase by 26 and 10–25% respectively. Finally, r*Ixs*S17 also inhibited blood clotting fXIIa (15 nM) by 18–33%, neutrophil elastase (22 nM) by 18–25%, pancreatic chymotrypsin (1.4 nM) by 10–27%, human thrombin (19 nM) by 28–34%, fIXa (311.4 nM) and pancreatic kallikrein (20 nM) by ~10%. Lastly, r*Ixs*S17 had no inhibitory activity against bovine thrombin (undefined) and pancreatic elastase (19 nM). Heat-inactivated r*Ixs*S17 did not inhibit serine proteases (inhibition rate = 0%) suggesting that its inhibitory activity is heat sensitive.

Next, we tested if proximity of the hexa-His-fusion tag to the RCL or its absence affected inhibitory activity of r*Ixs*S17 against selected proteases (Fig 3B). As shown, r*Ixs*S17 with N-terminal hexa-His-fusion tag had an 8.6% decrease in the inhibitory activity against trypsin. This suggests that beside the C-terminus domain that contains RCL, extension of the N-terminus region of the serpin might affect its inhibitory activity against trypsin. For both factor Xa and thrombin, inhibitory activity of the three constructs were similar. Since C-terminal histidine and non-tagged r*Ixs*S17 have equal inhibitory activity, either of them was used in our downstream assays.

**Fig 4:**
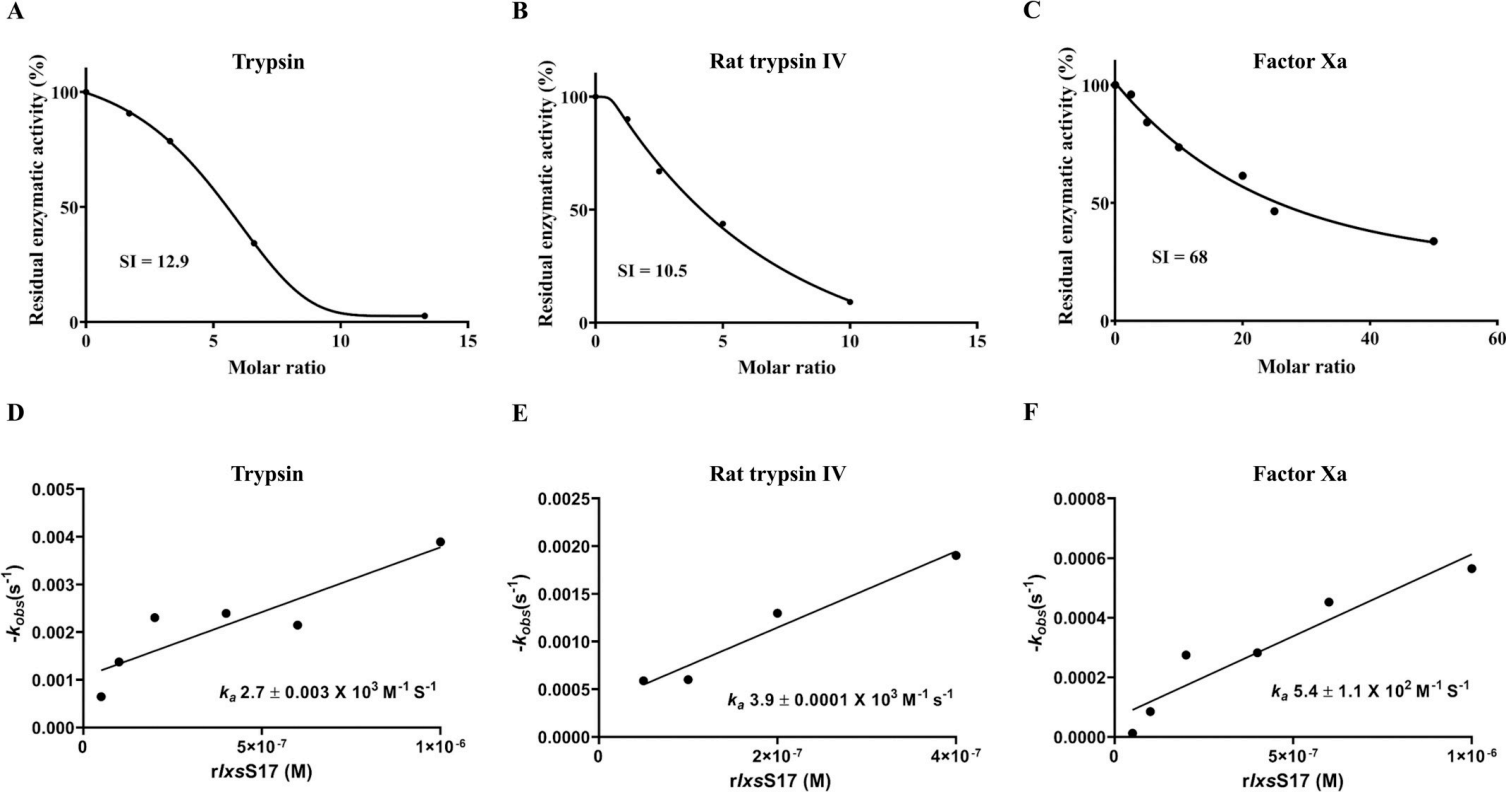
r*Ixs*S17 is a moderate inhibitor of trypsin, rat trypsin IV and factor Xa. Stoichiometry inhibition (SI) analysis calculated the amount of r*Ixs*S17 needed to inhibit one molecule of bovine trypsin (A), rat trypsin IV (B), and factor Xa (C). Various molar ratios of r*Ixs*S17 to proteases (0, 2.5, 5, 10, 20, 25, 50) were incubated for 15 min at 37°C with constant concentration of bovine trypsin (1.5 nM) or rat trypsin IV (2.0 nM) or factor Xa (13.9 nM). In the presence of appropriate substrates, residual enzymatic activity was measured and plotted against r*Ixs*S17: protease molar ratio. The SI was determined by extrapolating to the r*Ixs*S17: protease ratio where protease activity is zero (Y axis = 0). The inhibition rate (*k*_*a*_) of r*Ixs*S17 was determined against bovine trypsin (D), rat trypsin IV (E) and factor Xa (F). Different concentrations of r*Ixs*S17 (50, 100, 200, 400, 600 and 1000 nM) were incubated with constant amounts of bovine trypsin (14.6 nM), trypsin IV (12.5 nM) or factor Xa (13.9 nM) for different periods of time (0, 1, 2, 4, 6, 8, 10 and 15 min) at 37°C. The residual protease activity was measured and plotted against time to determine the pseudo-first order constant, *k*_obs_. Consequently, the second-rate constant (*k*_*a*_) was determined by the best fit line slope of the *k*_obs_ values that were plotted against r*Ixs*S17 concentration.

To determine the efficiency and rate at which r*Ixs*S17 inhibits pancreatic trypsin, trypsin IV, and factor Xa, stoichiometry of inhibition (SI) and association rate of constant (*k*_*a*_) were calculated (Fig 4). As shown, the SI (amount of r*Ixs*S17 needed to inhibit one molecule of protease) for C-terminal His-tagged r*Is*S17 against trypsin, trypsin IV, and factor Xa was estimated at 12.9, 10.5, and 68 respectively (Fig 4A–4C). The rate of r*Ixs*S17 (*k*_*a*_) inhibition of trypsin, trypsin IV, and factor Xa was 2.7 ± 0.003 x10^3^ M^-1^ s^-1^, 3.9± 0.0001 x10^3^ M^-1^ s^-1^, and 5.4 ± 1.1 x 10^2^ M^-1^ s^-1^, respectively (Fig 4D–4F).

The concentration of r*Ixs*S17 (1 μM) used in the inhibitory assays may not reflect the physiological levels of this protein in tick saliva, however, is at optimal concentration of a single tick salivary recombinant protein to be biologically active *in-vitro* or *ex-vivo* (1–6 μM) according to Chmelař et al., 2016 [52]. The reason is in the complex salivary mixture, this high concentration could be achieved by combination with numerous redundant proteins.

### r*Ix*sS17 interacts with both innate immune system effector proteases and regulatory protease inhibitors as revealed by protein-to-protein interaction analysis

**Fig 5:**
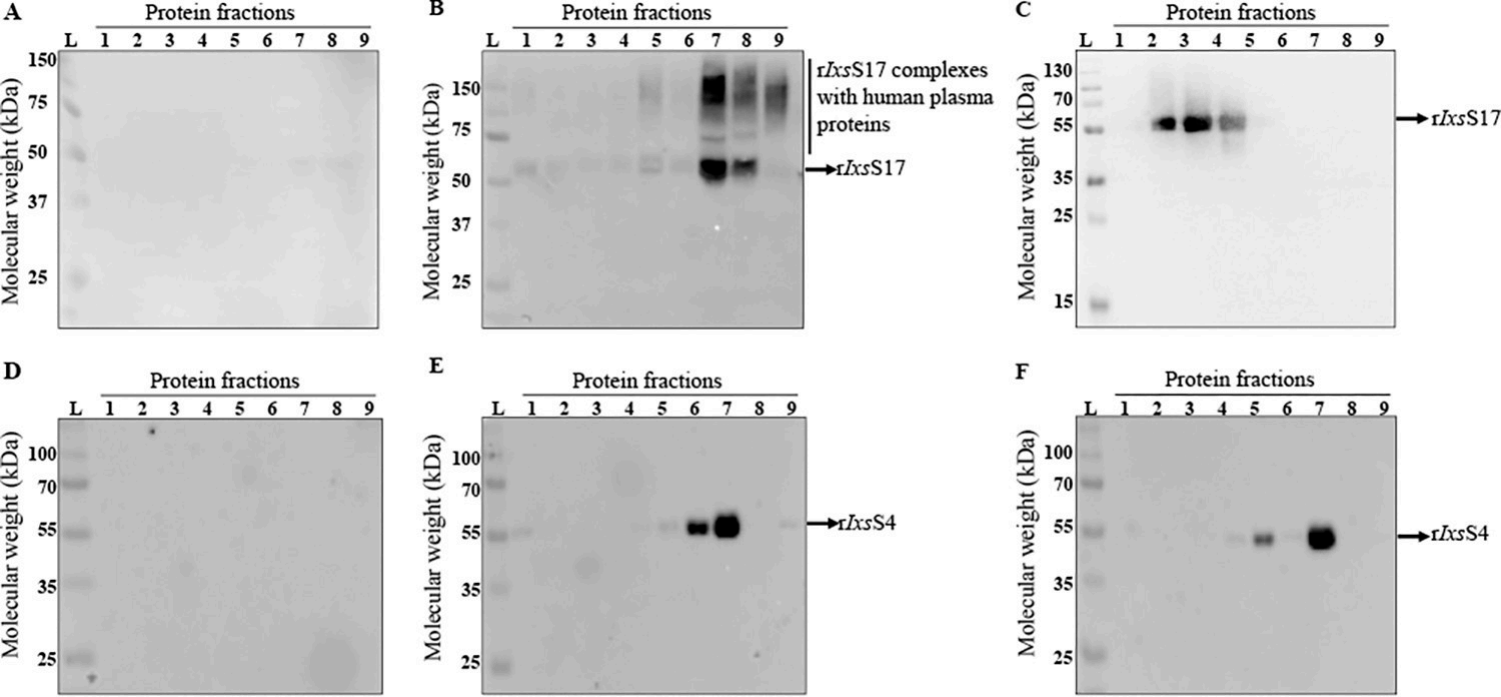
Protein-to-protein interaction using differential precipitation of proteins (DiffPOP) analysis reveals novel *Ixs*S17 functional insights. 25 μg of affinity purified r*Ixs*S17 (A-C) or r*Ixs*S4 (D-F) was incubated with human plasma in reaction buffer (20mM Tris-HCL and 150mM NaCl pH 7.4) overnight at 37°C. The reaction was stabilized using Phosphoprotein Kit- Buffer A and subjected to repeated precipitation (X10) using methanol and acetic solution (90% methanol to 1% acetic acid). Appropriately washed precipitates of each fraction were resolved on 10% SDS-PAGE and transferred onto PVDF membrane for western blot analysis using monospecific antibodies to r*Ixs*S17. A and D = human plasma only, B and E = human plasma mixed with r*Ixs*S17 or r*Ixs*S4, and C and F = r*Ixs*S17 or *rIxs*S4 alone. Ladder (L), Number (1–9) represents each fraction from differential precipitation. Please note that, fraction 10 for r*Ixs*S17 is not shown in this figure; however, its LC-MS/MS data analysis is available in S1 File.

Fig 5A–5C, and Table 1 and S1 File summarize the differential precipitation protein-protein interaction analysis of r*Ixs*S17 and human plasma proteins. Since low amounts of r*Ixs*S17 were detected in fractions 1–6, we pooled fractions 1–3, and 4–6 while fractions 7–10 where individually analyzed in LC-MS/MS analysis (S1 File). Next, we used PSOPIA (prediction server of protein-to-protein interactions; https://mizuguchilab.org/PSOPIA/) to analyze the interaction if NSAF (normalized spectral abundance factor) value was higher in r*Ixs*S17 and human plasma mixture compared to plasma only controls. The interactions that were predicted with more than 75% likelihood by PSOPIA were considered true (Table 1). This analysis confirmed substrate hydrolysis results and revealed novel insights that r*Ixs*S17 likely interacts with both effector proteases and regulatory protease inhibitors of the innate immune system (Table 1). Differential precipitation and PSOPIA analysis revealed that r*Ixs*S17 interacted with blood clotting system factors (f) II (prothrombin), fX, fXII, plasma kallikrein, and plasminogen alongside blood clotting regulatory protease inhibitors; antithrombin III (serpin), heparin cofactor II (serpin), alpha-2 macroglobulin (non-serpin inhibitor), and Kininogen-1 (non-serpin inhibitor) (Table 1). Similarly, r*Ixs*S17 interacted with complement system serine proteases, C1s, C2, and factor I alongside the complement system regulatory serpin, plasma protease C1 inhibitor (Table 1). Complement component C3, C4 and C5 were detected in the differential precipitation of proteins analysis (S1 File); however, PSOPIA predicted weak likelihood for interaction. We also found that r*Ixs*S17 interacted with non-protease blood clotting system proteins (fibronectin and fibrinogen), non-inhibitory serpins (angiotensinogen, and pigment endothelium derived factor), and non-proteases (haptoglobin and platelet endothelial aggregation receptor 1) (Table 1 and S1 File). Notably r*Ixs*S4 (XP_040066711.2 or XP_040066712.2), an inhibitor of trypsin that similar to *Ixs*S17 has basic amino residue (R) at its P1 site did not interact with human plasma in the differential precipitation protein-protein interaction (Fig 5D–5F). This finding confirmed that the r*Ixs*S17 and plasma protein-to-protein interactions were specific.

**Table 1:** Fast fractionation and LC-MS/MS analyses identification of human plasma proteins that interacted with r*Ixs*S17 and validation using *in silico* protein to protein interaction prediction PSOPIA software.

Accession	Description	Functional Classification	PSOPIA P2P prediction score
P00742	Coagulation factor X	Protease—Blood Coagulation	0.8573
P00748	Coagulation factor XII	Protease—Blood Coagulation	0.7918
P01042	Kininogen-1	Protease—Blood Coagulation	0.9505
H0YAC1	Plasma kallikrein	Protease—Blood Coagulation	0.8573
A8K9A9	Plasma kallikrein B	Protease—Blood Coagulation	0.8573
H0VJK2	Plasminogen	Protease—Blood Coagulation	0.891
P00734	Prothrombin	Protease—Blood Coagulation	0.996
P06681	Complement C2	Protease–Complement proteins	0.8204
P09871	Complement C1s subcomponent	Protease—Complement proteins	0.8054
P05156	Complement factor I	Protease—Complement proteins	0.8054
P01023	Alpha-2-macroglobulin	Protease Inhibitor—Blood Coagulation	0.7279
P01008	Antithrombin-III	Protease Inhibitor—Blood Coagulation	0.9794
P05546	Heparin cofactor 2	Protease Inhibitor—Blood Coagulation	0.9794
P05155	Plasma protease C1 inhibitor	Protease Inhibitor—Complement	0.9794
P01019	Angiotensinogen	Protease inhibitor—Non inhibitory Serpin	0.9695
P36955	Pigment epithelium-derived factor	Protease inhibitor—Non inhibitory Serpin	0.9794
P00739	Haptoglobin-related protein	Metal Binding Proteins	0.8442
Q5VY43	Platelet endothelial aggregation receptor 1	Receptor	0.7513

PSOPIA score of 0.75 to 1.0 represent likely protein to protein interactions (Murakami and Mizuguchi, 2014)

### *rIxs*S17 binds glycosaminoglycans (GAGs)

**Fig 6:**
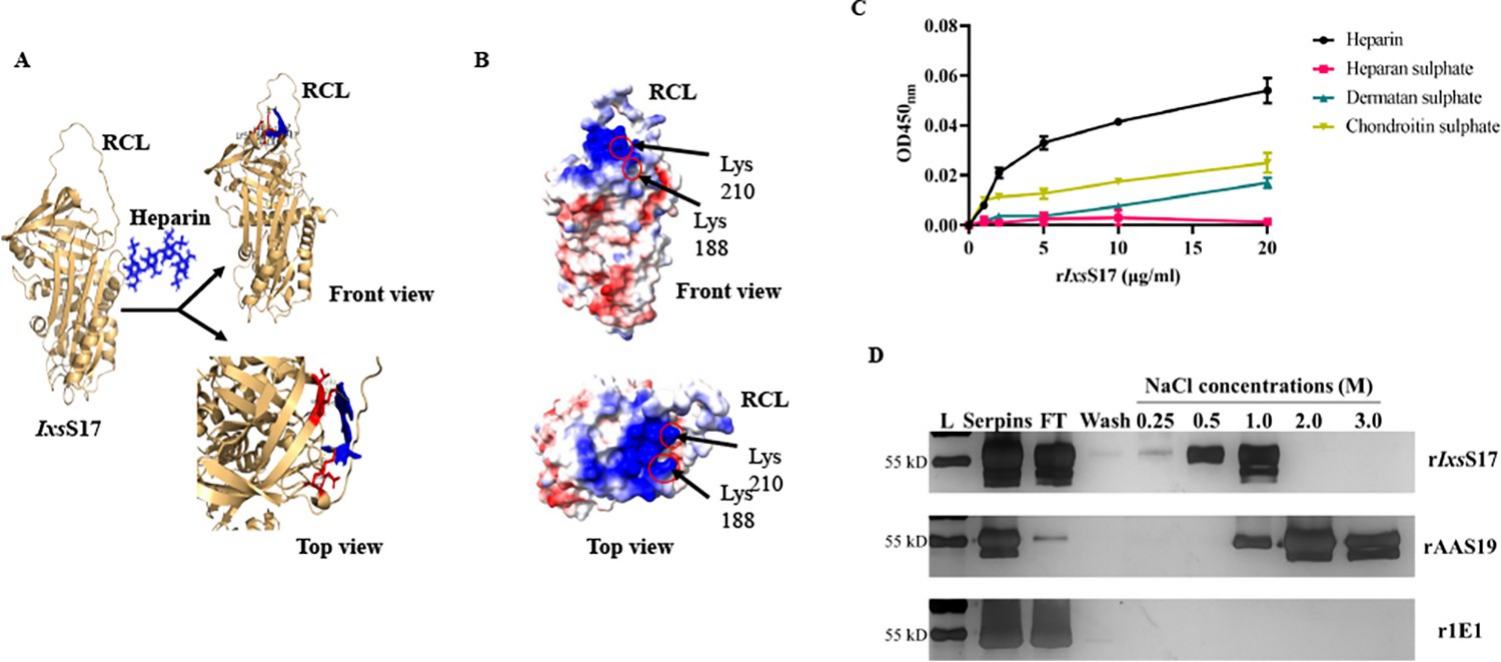
*Ixs*S17 binds heparin and the putative binding sites are located on the positive basic patch. (A) Comparative modeling of *Ixs*S17 secondary structure was predicted on Chimera X server and heparin binding predicted using Autodock Vina and Auto Dock Tools. Heparin ligand (in blue) was arranged accordingly to be flexible to rotate and to explore the most probable binding positions (in red) while the receptor was kept rigid. RCL = reactive center loop. (B) Two heparin binding sites at Lysine 188 and 210 are located on the positive basic patch (Electrostatic potential is color coded: positive is blue; neutral is white and negative is red). (C) Binding affinity of r*Ixs*S17 to 4 different GAGs: heparin (black circle), heparan sulphate (red square), dermatan sulphate (green triangle) and chondroitin sulphate (yellow upside-down triangle). The r*Ixs*S17 was added into 96-well microplates previously coated with different GAG at the concentration of 0, 1, 2, 5, 10 and 20 μg/mL. Binding was detected using HRP-conjugated antibody to the histidine tag and documented as *A*_450nm_. The data represent mean ± SEM from 3 biological replicates. (D) Silver staining of r*Ixs*S17, rAAS19 (positive control), and r1E1 (negative control) eluted from heparin column. Recombinant proteins (~300 μg) were applied to the heparin column. After washing, the proteins were eluted using a gradient concentration of NaCl (0.25–0.5–1.0–2.0–3.0 M). Serpins = the proteins before applying to the column, FT = Flow through.

Homology modeling predicted that *Ixs*S17 secondary structure, which was scored at Coulombic electrostatic values of -10 to 10 on ChimeraX server has a single basic positive patch located near the RCL (Fig 6A and 6B). Basic positive patch could potentially bind negatively charged ligands such as GAGs [53,54]. Consistently, docking analysis conducted by AutoDock Vina and ADT v1.5.4 demonstrated that the *Ixs*S17 secondary structure is likely to bind with heparin (Fig 6B). For the docking, nine poses were predicted and the result with the binding affinity of -12.6 Kcal/mol and the lower bound and upper bound RMSD as 0 were selected to be the best docked conformation. Further, the result generated was visualized by PyMOL, which shows that 5 amino acids of *Ixs*S17 (Asp185, Lys188, Lys210, Ser211, Thr212) were interacting with heparin; and out of 5 binding sites, 2 heparin binding sites (Lys 188 and 210) lies within the positively charged or basic patch which was predicted by Chimera X. Finally, we confirmed that r*Ixs*S17 has high binding affinity for heparin followed by chondroitin sulphate and dermatan sulphate (Fig 6C). However, it was interesting to observe that r*Ixs*S17 bound to heparin, but not to heparan sulphate (Fig 6C). It might be because of structural variations between heparin and heparan sulphate, such as the chain of heparan sulphate is generally longer, with higher molecular weight (30kDa) than heparin (15kDa). Furthermore, l-iduronic acid predominates in heparin while d-glucuronic acid represents most of the uronic acid found in heparan sulfate. This changes the structure configuration resulting into alteration in binding affinity. Most importantly, heparin is the complete modified version of heparan sulphate and contains highest negative charge density of any known biological macromolecule which will increase its binding affinity to the positive patch of r*Ixs*S17 [55]. The relative binding affinity to heparin was further determined showing that r*Ixs*S17 bound on the heparin column and was eluted at 0.25-1M of NaCl (Fig 6D). For positive control, *Ixs*S17 *A*. *americanum* homolog, rAAS19 which is known for high binding affinity to heparin and having 4 basic patches [35] was eluted at higher concentrations of NaCl (1-3M). The negative control r1E1 (KF990169) does not bind heparin and does not have a basic patch [45], therefore, came out in the flow-through.

### Binding of heparin significantly enhances anti-blood clotting effects of r*Ixs*S17

**Fig 7:**
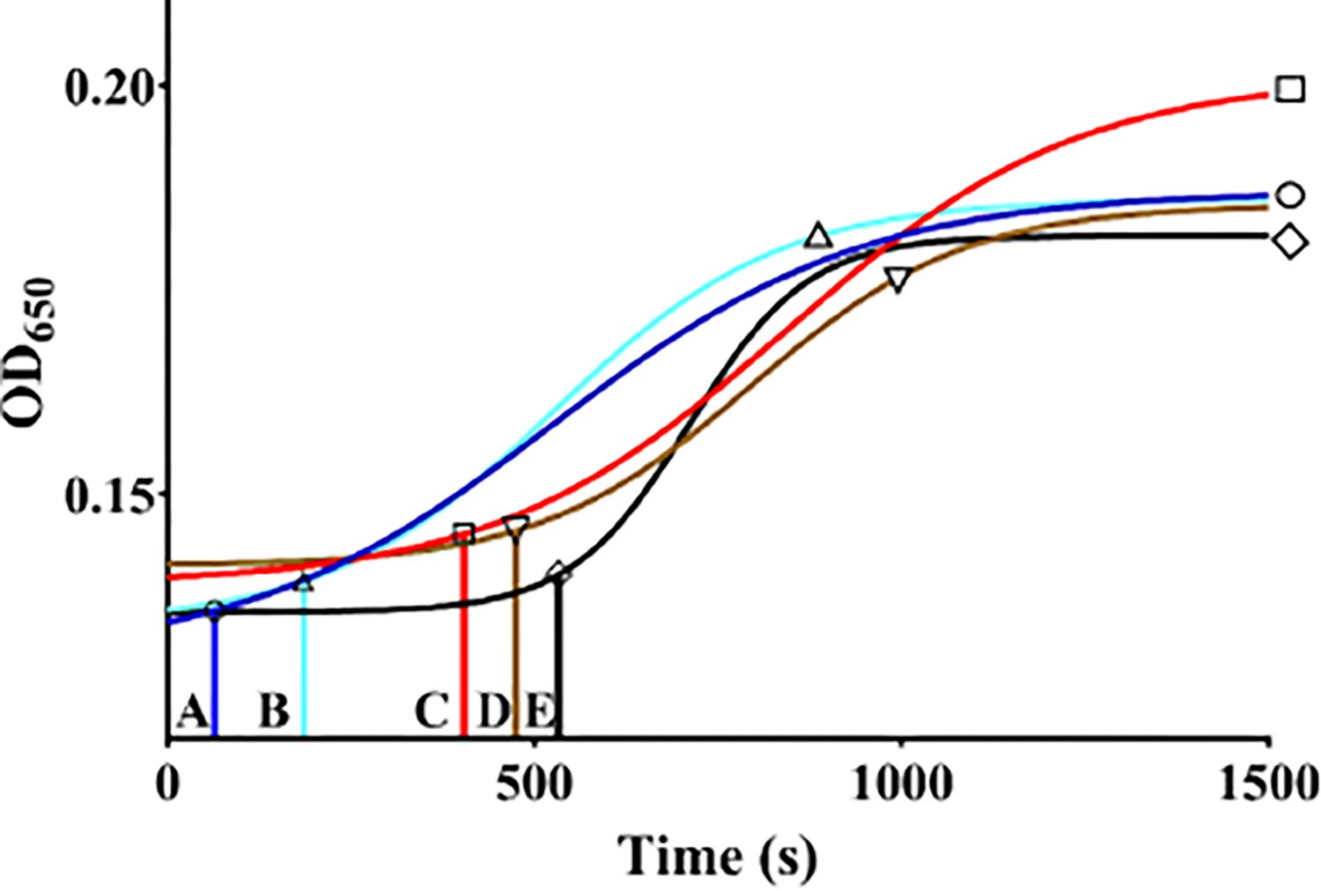
Heparin binding enhances r*Ixs*S17 anti-coagulant activity. Anti-blood clotting effects of heparin binding on r*Ixs*S17 was determined in the recalcification time assay. Universal coagulation reference human plasma was pre-incubated with (1) reaction buffer control, or (2) heparin only, or (3) r*Ixs*S17 only, or (4) r*Ixs*S17 and heparin pre-incubated together, or (5) r*Ixs*S17 and heparin pre-incubated separately. CaCl_2_ was added to trigger blood clotting and the reaction was monitored at *A*_650nm_ every 20s for 20 min. The *A*_650nm_ data were then fitted in the Sigmoidal dose-response lines: blue (buffer control), red (heparin only), green (r*Ixs*S17 only), black (r*Ixs*S17 and heparin pre-incubated together), and brown (r*Ixs*S17 and heparin pre-incubated separately). Clotting time was interpolated from the sigmoid line when *A*_650nm_ increases by 10% with 95% confident interval. Drop vertical lines A, B, C, D, and E = clotting time for buffer only (circle), r*Ixs*S17 only (triangle), heparin only (square), r*Ixs*S17 and heparin pre-incubated with plasma separately (upside-down triangle), r*Ixs*S17 and heparin pre-incubated with plasma together (diamond).

In preliminary studies, we empirically determined that 2 μM of r*Ixs*S17 delayed plasma clotting by more than 60 seconds compared to buffer control. Next, we tested if the combination of r*Ixs*S17 and 17 kDa heparin had synergistic anti-plasma clotting effect. As heparin is an approved blood clotting disorder therapeutic [56–58], it is interesting to note that pre-incubating plasma with the r*Ixs*S17 and heparin mixture significantly delayed plasma clotting up to 532.9 seconds compared to clotting time for buffer control (64.5 seconds), r*Ixs*S17 only (184 seconds), and heparin only (407 seconds) (Fig 7). It is also notable that plasma clotting was also delayed to 474 seconds when a reaction was assembled from plasma that was incubated separately with r*Ixs*S17 and heparin.

### r*Ixs*S17 inhibits complement activation via the mannose-binding lectin pathway and rescues *B*. *burgdorferi* from complement-mediated killing

**Fig 8:**
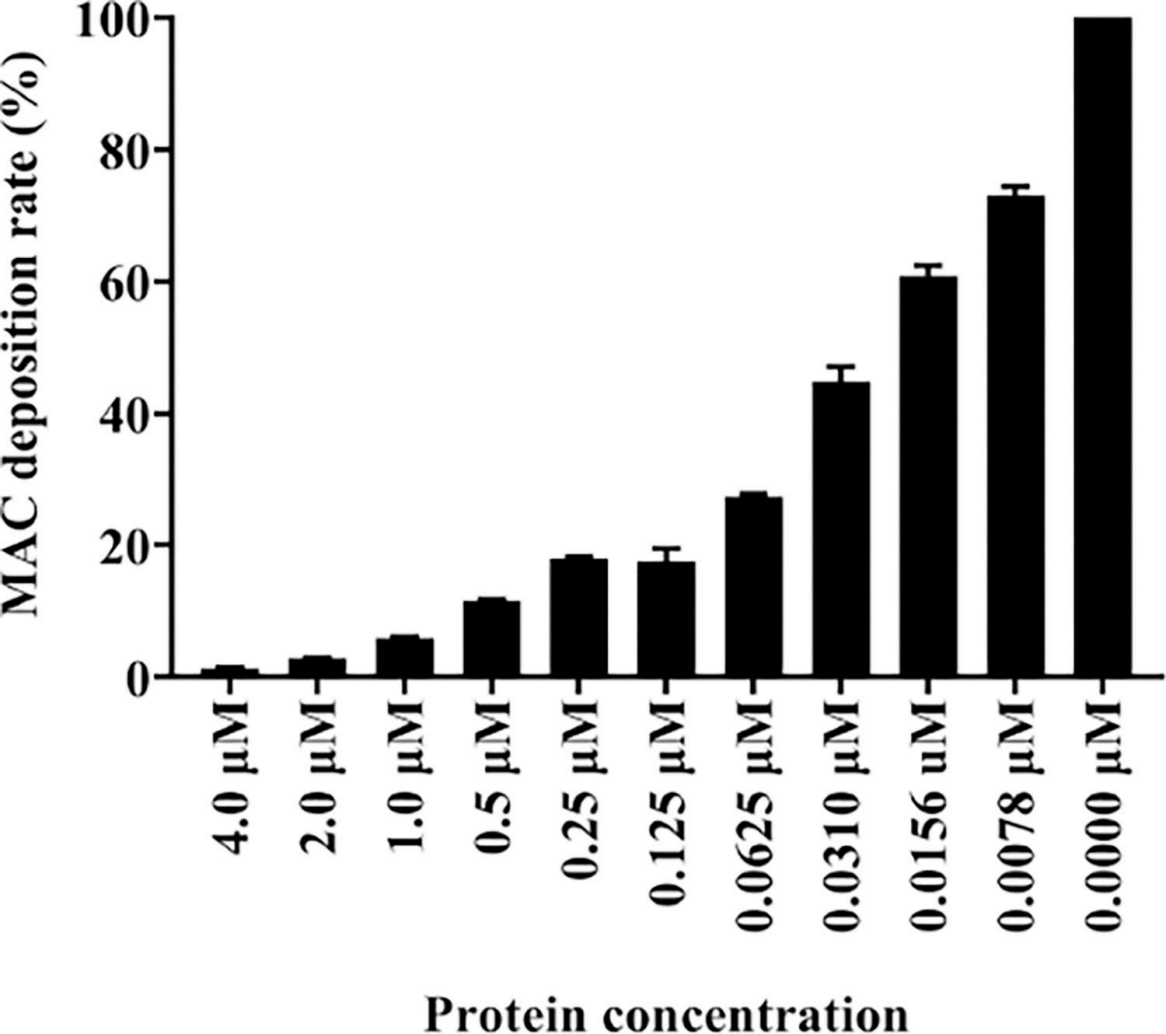
r*Ixs*S17 dose dependently inhibited membrane attack complex deposition of the Mannose-Binding Lectin pathway. A Blank, Negative control, Positive control or Positive control incubated with 2-fold-serial dilution of r*Ixs*S17starting from 4 μM were added to the Mannan binding lectin (MBL) pathway Kit and incubated at 37°C for 60 min. After the washing step, conjugate and substrate were subsequently added following the instructions of the manufacturer. Mac deposition rate was calculated using the formular: (Sample-NC)/(PC-NC) x100%, where NC is negative control and PC (or 0.0 μM of r*Ixs*S17) is positive control. Data is presented as percent inhibition of MAC deposition mean ± SEM calculated from 3 biological replicates.

Consistent with protein-to-protein interaction (*in silico* and *ex vivo*) showing that r*Ixs*S17 interacted with complement system serine proteases (C2, C1s and factor I), our data shows that *Ixs*S17 is an inhibitor of the complement system (Table 1 and Fig 8). We successfully used the WIESLAB complement system kit to independently assess three complement activation pathways. The results demonstrated that r*Ixs*S17 significantly inhibited deposition of the complement membrane attack complex (MAC) via the mannose-binding lectin (MBL) complement activation pathway and moderately via the classical and alternative complement activation pathways (S4 Table). In the initial screen, r*Ixs*S17 molar excess (4 μM) reduced MAC deposition by ~40, 62, and 99% via the classical, alternative and MBL pathway, respectively (S4 Table). Moreover, we found that r*Ixs*S17 dose dependently reduced by more than 55% MAC deposition through 31 nM of r*Ixs*S17 (Fig 8). In this study, dose response analysis was not done for the classical and alternative complement activation pathway.

**Fig 9:**
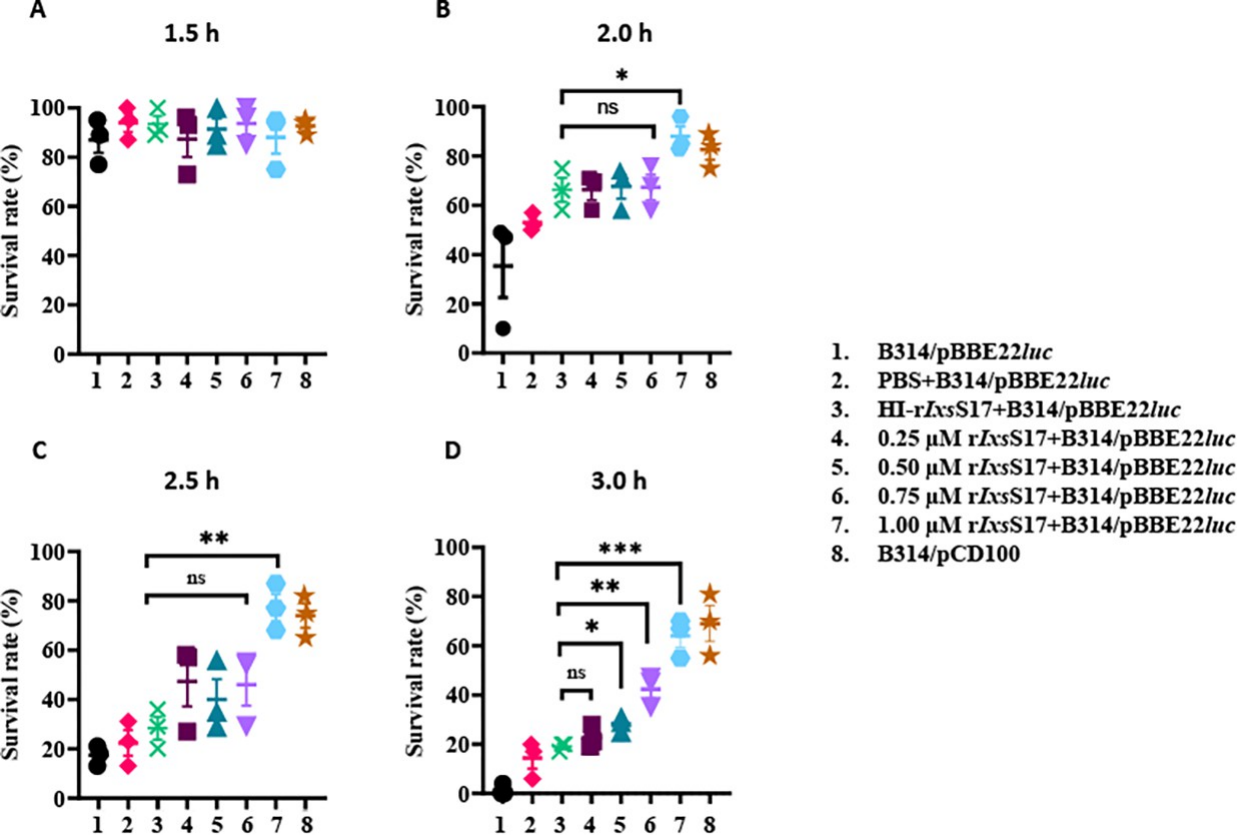
r*Ixs*S17 impaired complement mediated killing of *Borrelia burgdorferi*. Normal human serum (NHS) was pre-incubated with serial dilutions of *rIxs*S17 (0.25, 0.5, 0.75, and 1.0 μM) or heat-inactivated *rIxs*S17 (1.0 μM) or Phosphate buffered saline (PBS) at 37°C for 30 min prior to addition of 85 μl of 10^6^ cells/mL of *B*. *burgdorferi* B314/pBBE22*luc* (complement sensitive strain) and incubated in a bio-shaker at 32°C, 100 rpm. NHS incubated with *B*. *burgdorferi* B314/pPCD100 (complement resistant strain) were used as positive control. Survival rates of *B*. *burgdorferi* were assessed at 1.5 h (A), 2 h (B), 2.5 h (C) and 3 h (D) post incubation. Data represents mean ± SEM of 3 biological replicates. Statistical significance was evaluated using *t* test in GraphPad Prism 9 (ns: no significance, *:P value ≤ 0.05, **: P value ≤ 0.01, ***: P value ≤ 0.001). Negative control: black circle, PBS: red diamond, HI-*rIxs*S17: green cross, 0.25 μM *rIxs*S17: maroon square, 0.5 μM *rIxs*S17: green triangle, 0.75μM *rIxs*S17: purple upside-down triangle, 1μM *rIxs*S17: blue hexagon, Positive control: orange star.

*B*. *burgdorferi* can activate three complement pathways resulting in several host defense mechanisms that include: opsonization, phagocyte recruitment, priming of the adaptive immune system, and bacteriolysis [59,60]. Serum-mediated bacteriolysis has been used to test the sensitivity of LD spirochetes to normal human serum [61,62]. Next, we tested if r*Ixs*S17 was able to rescue *B*. *burgdorferi* from complement-mediated killing *in vitro* (Fig 9A–9D). Consistent with its inhibitory effect against complement activation (Fig 8), r*Ixs*S17 dose dependently rescued the complement sensitive *B*. *burgdorferi* strain B314/pBBE22*luc* from complement killing. At 1 h post incubation, *B*. *burgdorferi* survival rates ranged from 73–100% and were not different among the tested groups (Fig 9A). At 2 and 2.5 h, only the positive control (complement resistant *B*. *burgdorferi* strain B314/pCD100) and the 1μM r*Ixs*S17 groups had higher survival rates than negative control, heat-inactivated r*Ixs*S17 and PBS (protein buffer control) (Fig 9B and 9C). At 3 h of incubation, 0–14% of the negative control survived while survival increased to 19–21% (22 ± 2.7%), 25–31% (28 ± 1.8%), 35–47% (42 ± 3.7%) and 55–70% (64 ± 7.9%) in the presence of 0.25, 0.5, 0.75 and 1 μM r*Ixs*S17, respectively (Fig 9D). The positive control had survival rates of 43–67% (56 ± 12.1%). In heat-inactivated normal human serum, survival rates of all the tested groups ranged from 97–100%.

### Co-injecting r*Ixs*S17 enhances *B*. *burgdorferi* colonization of C3H/HeN heart and skin (ear)

Next, we tested if r*Ixs*S17 supported *B*. *burgdorferi* colonization of the C3H/HeN Lyme disease mouse model. We initially inoculated six groups of mice (4 mice per group) with BSK-II medium (negative control), *B*. *burgdorferi* in BSK-II (positive control) and *B*. *burgdorferi* mixed with various amounts of r*Ixs*S17 (1, 2, 5 and 10 μM). *B*. *burgdorferi* infection was confirmed in all treated groups by *in-vitro* cultivation and conventional PCR (S5 and S6 Tables). Spirochete burden in the ear, heart, joint and bladder tissues did not show significant differences between the groups (S4 Fig). However, we observed a pattern of *B*. *burgdorferi* load decreasing with increasing concentration of r*Ixs*S17 in the heart and bladder (S4 Fig). Moreover, IgG antibody titer against *B*. *burgdorferi* was statistically lower in the higher r*Ixs*S17 concentration groups (S5 Fig).

**Fig 10:**
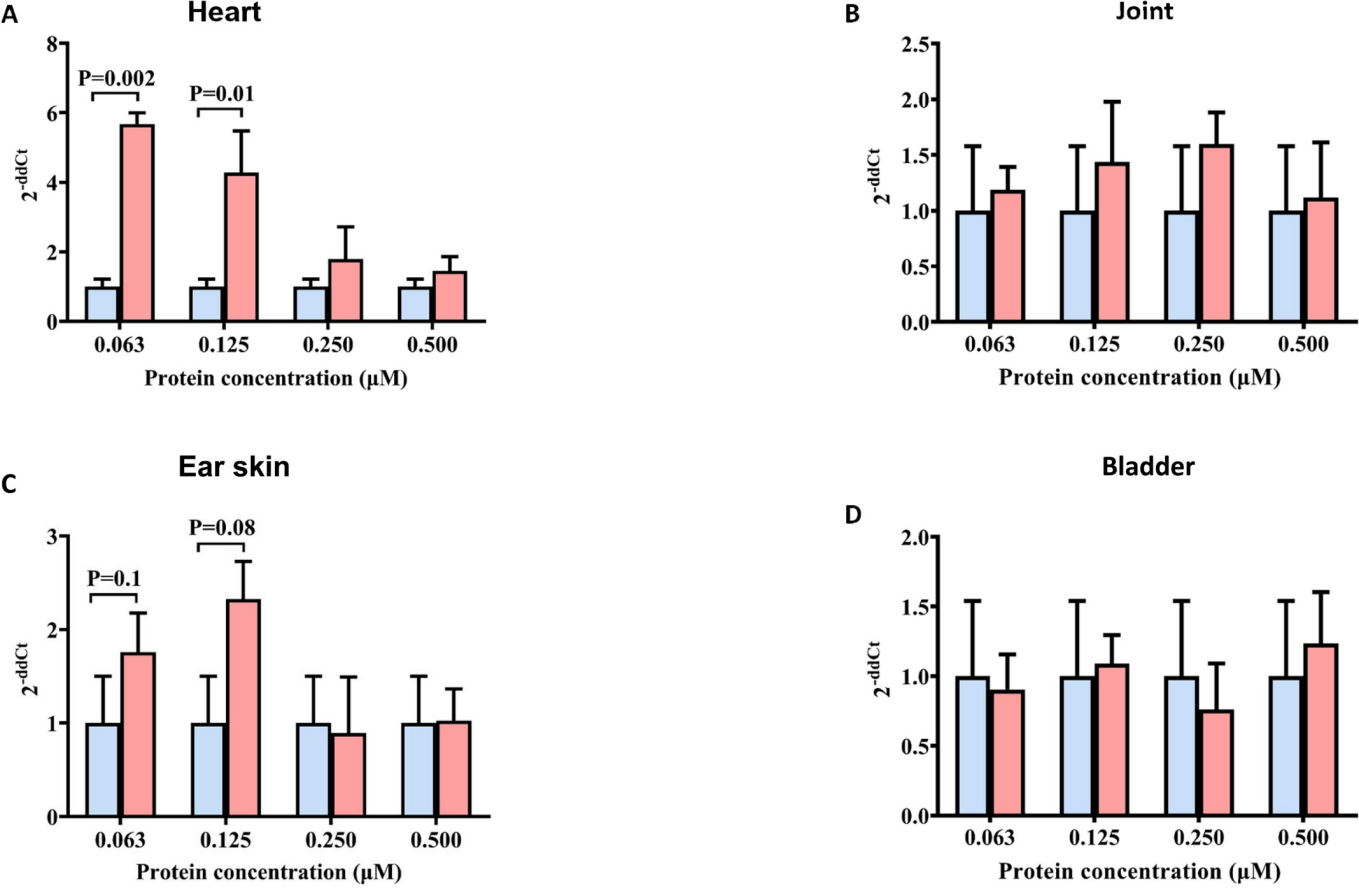
Mouse groups co-inoculated with low dose of r*Ixs*S17 have higher *B*. *burgdorferi* load in organs than high dose injected mice. Four mice/group were inoculated with *B*. *burgdorferi* only (10^4^ cells) mixed with or without various amounts of r*Ixs*S17 (0.060, 0.125, 0.250, 0.500 μM). At 21 days post inoculation, *B*. *burgdorferi* burden in mouse heart (A), joint (B), ear (C) and bladder tissues (D) was quantified by real-time qPCR method. The data were presented as fold change of the r*Ixs*S17 treated groups in comparison with *B*. *burgdorferi* group (2 ^-ΔΔCt^ = [(C_t_ Flab—C_t_ β-Actin) *B*. *burgdorferi*-r*Ixs*S17 co-injected group—(C_t_ Flab—C_t_ β-Actin) Bb only group]). Blue: *B*. *burgdorferi* only group, Red: *B*. *burgdorferi*-r*Ixs*S17 co-injected group.

We decided to repeat the experiment with reduced concentrations of r*Ixs*S17: 0.06, 0.125, 0.25 and 0.5 μM (Fig 10). We show that the spirochete load in the heart tissue of r*Ixs*S17 injected mice with 0.06 and 0.125 μM of r*Ixs*S17 was 5.7 and 4.3 folds significantly higher than *B*. *burgdorferi* only group (Fig 10A). Likewise, there is an apparent trend (P < 0.1) that the spirochete load in 0.06 and 0.125 μM r*Ixs*S17 injected mice was 1.8 and 2.3 folds higher than the *B*. *burgdorferi* only group in ear tissues (Fig 10C). Similarly, there is an apparent high *B*. *burgdorferi* load in joints of mice that were co-injected with 0.125 and 0.25 μM r*Ixs*S17 than control (Fig 10B) and there is no apparent difference in the bladder (Fig 10D). To determine whether high concentrations of r*Ixs*S17 affected the survival of *B*. *burgdorferi* in the inoculum, we incubated *B*. *burgdorferi* with 0, 0.06, 0.125, 0.25, 0.5, 1, 5, and 10 μM of r*Ixs*S17 *in vitro*. This analysis revealed r*Ixs*S17 did not have a negative effect on *B*. *burgdorferi* in culture as spirochete survival ranged from 95–98% up to 24h of observation (S7 Table).

**Fig 11:**
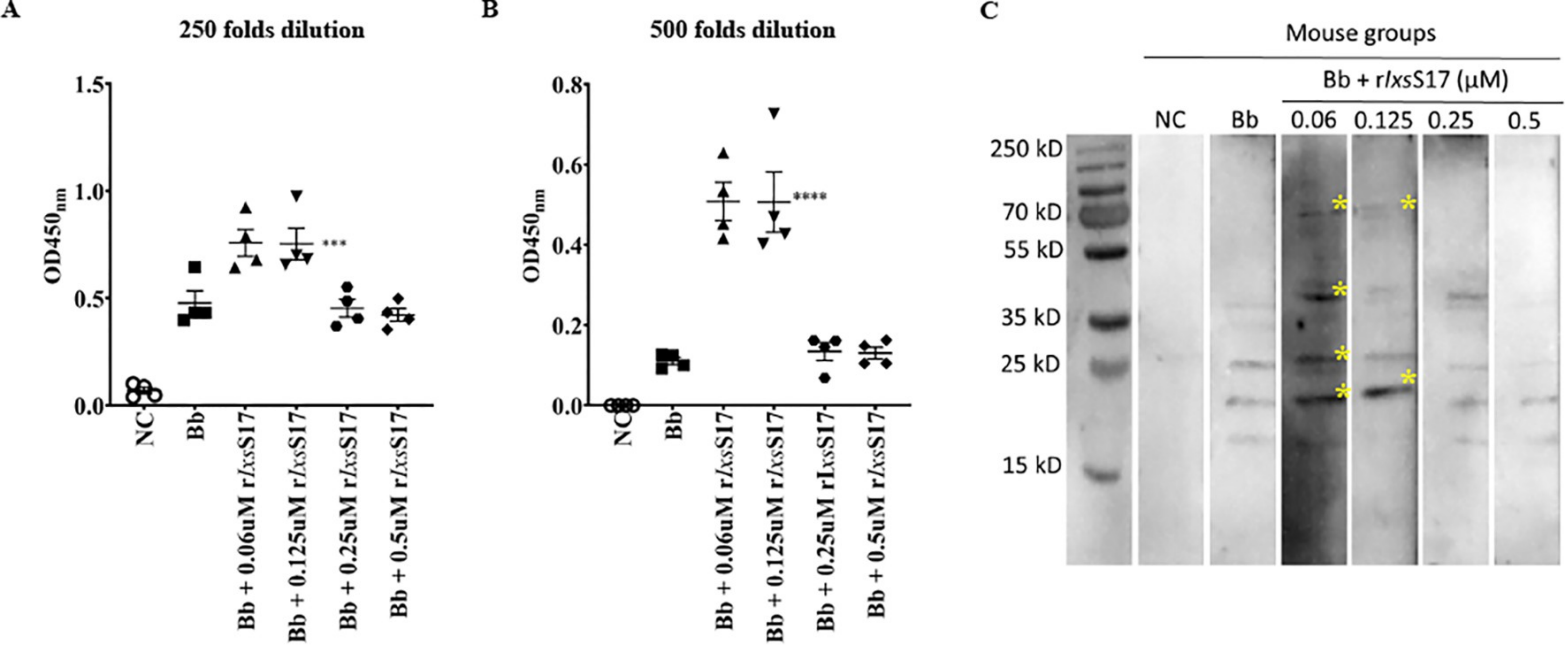
Mouse groups co-inoculated with low dose of r*Ixs*S17 have higher IgM titers compared to high dose injected mice. ELISA plates previously coated with *B*. *burgdorferi* crude antigen (200 ng/well) were tested with mouse sera that diluted at 1:250 (A) and 1:500 (B). IgM titers were determined using anti-mouse IgM monospecific antibody conjugated with HRP and absorbance were read at 450 nm. The data were presented as mean ± SEM; each dot is individual mouse. NC = negative control; Bb = *B*. *burgdorferi*. Statistical significance was evaluated using *t* test in GraphPad Prism 9 (***:P value ≤ 0.001, ****: P value ≤ 0.0001). (C). Western blot analysis of *Bb* lysate (2 μg) incubated with antisera from mice (diluted to 1:200) and anti-mouse IgM antibody-HRP conjugate (diluted to 1:5000). Images were taken at 18 seconds of exposure. Asterisks indicate extra and intense bands detected in mice challenged with *Bb* plus 0.06 and 0.125 μM r*Ixs*S17.

It is also notable that IgG titers to *B*. *burgdorferi* lysate antigen detected in ELISA of the *B*. *burgdorferi* control and r*Ixs*S17-treated groups were not statistically different (S6 Fig). However, IgM titers of the 0.06 and 0.125 μM r*Ixs*S17 co-injected groups were significantly higher than 0.25 and 0.50 μM of r*Ixs*S17 co-injected groups. Interestingly the IgM antibody of mice that were co-injected with 0.25 and 0.50 μM of r*Ixs*S17 did not show any significant difference with *B*. *burgdorferi* control mice (Fig 11A and 11B). Furthermore, immune sera of 0.06 and 0.125 μM r*Ixs*S17 co-inoculated mice bound multiple bands on western blots of lab cultured *B*. *burgdorferi* lysate (Fig 11C).

## Discussion

This study provides data showing that *I*. *scapularis* serpin (*Ixs*S) 17 regulates key functions that are important to tick feeding and *B*. *burgdorferi* colonization of the host. It builds on previous studies done by our lab that characterized the *A*. *americanum* tick serpin 19 as the only tick serpin that has its functional domain RCL perfectly conserved (100%) in all tick species as per available data [34,35]. Like most parasites, ticks have the propensity for encoding redundant molecular systems. For tick serpins, it is common for multiple paralogs or isoforms showing more than 70% amino acid identity being transcribed by the same tick species [34,40,63] suggesting redundancy. Thus, the finding that the *Ixs*S17 amino acid sequence does not show any matches to other *I*. *scapularis* serpins with more than 50% amino acid identity suggests that this protein represents a non-redundant tick serpin. It is notable that except for *D*. *silvarum* which encodes for two homologs to *Ixs*S17 with more than 77% amino acid identity, 12 other tick species encoded single serpin homologs to *Ixs*S17, with the RCL being 100% conserved. It is important to note that the two *Ixs*S17 homologs in *D*. *silvarum* have the same RCL and the difference is limited an 11 amino acid deletion. We would like to note while amino acid sequence of *Ixs*S17 suggested no redundancy, we are unable to know in this study if *Ixs*S17 is also not functionally redundant.

The RCL plays an important role in the inhibitory functions of serpins [44], and thus it is not surprising that the functional roles of *Ixs*S17 is similar to its homologs in *A*. *americanum*, AAS19 [35], *R*. *microplus*, RmS15 [37,38], and recently *I*. *ricinus*, Iripin-8 [64,65]. Collectively, substrate hydrolysis and protein-to-protein interaction data in this study indicate that *Ixs*S17 is an inhibitor of innate immunity effector proteases associated with inflammation, nociception (pain sensing), hemostasis, and complement innate immune defenses all of which must be blocked by ticks to feed and transmit tick-borne pathogens. In addition to food digestion [66], pancreatic trypsin which was highly inhibited by r*Ixs*S17 is also found in blood circulation, accelerates blood clotting in the presence of calcium ions, pro-thromboplastic lipid, factor V, VII, and X [67], and it is also the major activator of protease-activated receptor 2 (PAR2) that initiates inflammation signaling [68]. Likewise, trypsin IV, also highly inhibited by r*Ix*S17 is associated with signaling of cutaneous local inflammation and nociception by activation of PAR2 on cutaneous neurons [69]. Cathepsin G regulates the inflammatory responses by stimulating production and maturation of cytokines and chemokines and controls the functional state of immune cells [70]. It is interesting to note that like *I*. *ricinus* Iripin-8 and *A*. *americanum* AAS19 [35,65], r*Ixs*S17 inhibited plasmin. At a glance, *Ixs*S17 inhibition of plasmin is counterintuitive because plasmin is known for degradation of fibrin clots and preventing platelet aggregation by cleaving PAR1 [68,71], which will benefit tick feeding. However, plasmin has additional functions on inflammation and wound healing by directly interacting with various cell types including leukocytes (monocytes, macrophages, and dendritic cells) and cells of the vasculature (endothelial cells, smooth muscle cells) as well as soluble factors of the immune system and components of the extracellular matrix [72,73]. In these interactions, plasmin contributes to inflammation and thus, its inhibition by *Ixs*S17 will help tick feeding through prevention of inflammatory processes. While substrate hydrolysis reveals that r*Ixs*S17 weakly inhibited human thrombin and did not inhibit bovine thrombin, our protein-to-protein interaction data show that this protein interacted with thrombin (or blood clotting factor II) similar to its homologs; AAS19, Iripin-8, and Rm-15 [38,65,74].

Our protein-to-protein interaction data also revealed functional insights of r*Ixs*S17. The finding that r*Ixs*S17 interacted with both effector proteases and regulatory protease inhibitors of the innate immune system is intriguing because serpins are known for their role in inhibiting functions of effector serine and cysteine proteases [44]. Thus, it is surprising to note that r*Ixs*S17 interacted with both effector proteases and serpin regulators of the blood clotting pathway, antithrombin III (AT), heparin cofactor II (HCII), kininogen-1 and protease C1 inhibitor (C1 inhibitor) of the complement system. Glycosaminoglycans (GAGs) such as heparin [75] serve as cofactors to enhance the activity of some mammalian serpins such as AT [76,77] and HCII [78–80]. Comparative secondary structure modeling predicted basic patches in *Ixs*S17, which were confirmed as functional heparin binding sites using GAG plate binding and heparin column assays. As *Ixs*S17 and host serpins (AT and HCII) bind heparin, it is potentially possible that the observed interaction between *Ixs*S17 and host serpins was because of heparin serving as a bridge between *Ixs*S17 and host serpins. Likewise, as C1 inhibitor is heavily glycosylated [81,82], we hypothesize that *Ixs*S17 may interact with the C1 inhibitor by binding onto GAGs linked to this protein. It is important to also note that *in silico* analysis predicted direct interactions between r*Ixs*S17 and protease inhibitors with more than 95% chance. These observations warrant further investigations.

The function of serpins is effected by mechanical disruption of the target protease that starts with the target protease being trapped at P1 residue in the RCL [44]. Our results demonstrated that positioning the histidine fusion tag at the amino-terminus end and not the C-terminus end reduced the mechanical efficiency of r*Ixs*S17 which was restored by cleaving off the Histidine tag. The observed stoichiometry inhibition of r*Ixs*S17 against trypsin, rat trypsin IV, and factor Xa were high and not close to the ideal 1:1 serpin-to-target protease ratio. These findings could be explained by evidence some of the serpins such as blood clotting regulatory serpins antithrombin III and heparin II that require binding of glycosaminoglycans (GAGs) to enhance their inhibitory potency [58]. Similarly, we have shown that heparin binding potentiated functions of AAS19, the *Ixs*S17 homolog in *A*. *americanum* [74]. Likewise in this study, we determined that the putative basic patch in *Ixs*S17 comparative tertiary structure was functional and bound GAGs including heparin, an approved therapeutic against blood clotting disorders [56,71]. Consistent with AAS19 [74], heparin binding by r*Ixs*S17 significantly increased its anti-coagulation activity. The finding that heparin binding potentiated the function of r*Ixs*S17 suggests that when tick injects this protein into the host, native *Ixs*S17 binds GAGs to enhance its anticoagulant functions.

Our protein-to-protein interaction findings showing that r*Ixs*S17 interacted with complement system proteases. r*Ixs*S17 likely blocks complement activation by potentially interfering with complement component C2 (associated with classical and MBL pathway), C1s (classical pathway), and factors I (alternative pathway). The evidence led us to investigate the effect of this protein on complement activation. Complement system activation can be initiated via binding of specific antibodies (Classical pathway), mannose binding lectins (MBL pathway) or small-scale activation of complement component C3 (Alternative pathway) [59,60]. *B*. *burgdorferi* can activate all 3 complement pathways and results in direct complement-mediated killing of the spirochete [83]. Moreover, Schuijt et al., [84] showed that the MBL pathway is of paramount importance in the eradication of *B*. *burgdorferi*. Here, we showed that r*Ixs*S17 facilitated *B*. *burgdorferi* survival and promoted localization via inhibition of MBL pathway. By inhibiting the deposition of MAC in MBL pathway, r*Ixs*S17 rescued *B*. *burgdorferi* from complement-mediated killing *in vitro*.

Prompted by evidence that *Ixs*S17 is highly secreted by *B*. *burgdorferi* infected nymphs [25] within 24-48h of tick feeding, an open-window for transmission of LD agent, suggested that this protein is important to the transmission of *B*. *burgdorferi*. Kotál *et al*., [65] reported that Iripin-8 significantly influenced nymphal *I*. *ricinus* feeding but did not promote *B*. *afzelii* transmission as revealed by RNAi silencing. Here, we took a different approach and assessed the effect of co-injecting C3H with r*Ixs*S17 and *B*. *burgdorferi* and show that this protein promoted colonization of C3H mice. r*Ixs*S17 co-inoculation increased *B*. *burgdorferi* loads in heart and ear tissue, but not distal tissues like joint and bladder of C3H/HeN mice at 21 days post inoculation. This finding is relatively in agreement with *in vivo* experiment of Coumo *et al*., [85] that MBL deficiency mice had higher antibody titers and harbored significantly higher *B*. *burgdorferi* in skin tissue than deeper tissue (heart, joint and bladder) at 14 days post inoculation. Interestingly, our results showed that r*Ixs*S17 effects on *B*. *burgdorferi* localization of mouse tissue is inverse dose dependent as revealed by *B*. *burgdorferi* load and IgM titers being higher in mice that were injected with low dose (0.06 and 0.125 μM) than high dose (0.25, 0.5, 1, 2, 5 and 10 μM) of r*Ixs*S17. According to a study of Fikrig *et al*., [86], IgM to *B*. *burgdorferi* whole cell in infected mice peaks at day 14 post inoculation while IgG increases continuously even at day 180 after infection. Since IgG response to *B*. *burgdorferi* is much later during infection, it would explain why IgG titer in our experiment which we detected at day 21 (3 weeks) post inoculation did not show significant difference.

Although actual amount of *Ixs*S17 that the tick injects into the mammalian host during feeding is unknown, it is estimated to be in picomolar range. Thus, it makes logical sense that low concentrations of r*Ixs*S17 supported *B*. *burgdorferi* colonization and vice versa for the high dose. We hypothesize that the effects of low dose r*Ixs*S17 used in this study better resemble the dose of the native protein in tick saliva. It will also be interesting to further investigate if the low spirochete load in mice that received the high dose of r*Ixs*S17 was due to direct toxicity of this protein to *B*. *burgdorferi* or that the high dose over stimulated the innate immune system leading to clearance of the spirochetes.

LD control and prevention is challenging. The rise and spread of LD, and the fact that individuals can get LD more than once when bitten by an infected tick requires the development of novel effective vaccine against this vector-borne disease [87]. The search for vaccine target antigens is shifting from the pathogen toward tick molecules, with the purpose of reducing tick density and *B*. *burgdorferi* infection among tick population and blocking the transmission of LD agents [17,87,88]. In line with this, our data show that *Ixs*S17 is an important protein in both tick feeding and *B*. *burgdorferi* colonization of the host, and it represents a possible target antigen in vaccines to prevent transmission of tick-borne disease agents. The mechanism by which *Ixs*S17 protected *B*. *burgdorferi* from host innate immune response warrants future investigations.

## Materials and methods

### Ethics statement

The use of animals strictly followed the animal use protocol approved by Texas A&M University Animal Care and Use Committee under the number IACUC 2020–0089.

### Phylogeny and comparative sequence analysis

Amino acid sequence of *Ixs*S17 was obtained from NCBI protein database (Accession # EEC18973.1) and blasted using the protein-protein BLAST (blastP) tool (non-redundant protein sequences (nr) and transcriptome shotgun assembly proteins (tsa_nr) database), resulting in 12 *Ixs*S17 homolog sequences from different tick species. A *homo sapiens* antithrombin (Accession # CAA48690.1) was used as the out group in phylogenetic analysis of the sequences using MEGA X software [89]. Pairwise sequence alignment analysis between *Ixs*S17 and its homologs was used to determine protein identities. Based on the multiple sequence alignment analysis, the best protein model prediction and overall mean distance calculation, a phylogenetic tree was generated using maximum likelihood statistical method, bootstrap 1000 replicates, Le_Gascuel_2008 model and Gamma distributed with invariant sites (G+I) [90]. In previous studies, we showed that *Ixs*S17 sequence was likely not redundant because its functional domain RCL did not show amino acid identity of more than 55% to other *I*. *scapularis* serpins [34,35]. To confirm these analyses, we compared the amino acid sequence of *Ixs*S17 RCL to other *I*. *scapularis* serpin RCLs that were annotated in the recently updated *I*. *scapularis* genome [43]. Briefly, the extracted RCL regions from previously published serpins [40] were used as a query database to search each of the 34,235 protein sequences from the current *I*. *scapularis* reference genome (ASM1692078v2) using DIAMOND blastp v2.0.15 [91]. Each of the proteins with a positive hit to the RCL query was manually curated to confirm that it is a serpin with a complete RCL region. Finally, RCL regions from each of the confirmed *I*. *scapularis* serpins was compared to *Ixs*S17 RCL using Needleman-Wunsch Global Alignment tool from NCBI.

The protein signal peptides and subcellular localization of *Ixs*S17 were predicted using the software SignalP 6.0 (https://services.healthtech.dtu.dk/service.php?SignalP-6.0) and deepLoc 2.0 (https://services.healthtech.dtu.dk/service.php?DeepLoc).

### Expression of recombinant (r) *Ixs*S17

Expression of mature r*Ixs*S17 (without signal peptide) was done in *Pichia pastoris* using the pPICZαA yeast expression plasmid as described [35, 92]. The coding domain for mature *Ixs*S17 nucleic acid sequence was retrieved from GenBank (Accession # EEC18973.1) and optimized for expression in *P*. *pastoris*. Two r*Ixs*S17 expression constructs with His-fusion tag placed at amino- or carboxyl-terminus were custom synthesized and cloned into pPICZαA (Biomatik, Wilmington, Delaware). To facilitate cleaving off the His-fusion-tag, the Tobacco etch virus (TEV) protease cutting site (ENLYFQG) was inserted between His-fusion tag and the *Ixs*S17 coding sequence. The TEV protease was produced in house (see below).

Routinely, r*Ixs*S17 expression plasmid was transformed into *P*. *pastoris* X33 (Thermo Fisher Scientific, Hanover Park, IL, USA), cultured at 28°C in a bio-shaker (MaxQ 400, Thermo Fisher Scientific), and expression of r*Ixs*S17 induced by feeding cultures daily with 5% methanol as a carbon source. For large scale expression (1 L batches), cultures were incubated for 5 days. The pPICZαA yeast expression plasmid secretes the recombinant protein into culture media. Thus, spent media was collected and r*Ixs*S17 was precipitated out by ammonium sulfate saturation (525 g/L) at 4°C overnight with stirring. Subsequently, precipitated r*Ixs*S17 was dialyzed against His-tag affinity column binding buffer (100 mM Tris, 500 mM NaCl, 5mM imidazole, pH 7.4) and processed for affinity purification using the Hi-Trap Chelating HP column (Cytiva, Marlborough, MA, USA) under native conditions. Affinity purification was then confirmed by standard sodium dodecyl sulfate-polyacrylamide gel electrophoresis (SDS-PAGE), silver staining using the Pierce Silver Stain Kit staining kit (ThermoScientific, USA), and western blotting analysis using the mouse monoclonal anti-Histidine antibody (GenScript, Piscataway, NJ) to determine purity. The concentration of r*Ixs*S17 was determined using a bicinchoninic acid (BCA) kit (ThermoScientific, USA). The protein was stored at -80°C until use.

### Cleaving off the histidine tag from affinity purified r*Ixs*S17

The TEV protease was produced in house using expression vector MBP-TEVcs (ENLYFQ/G)-His6-TEVΔ (220–242)-R5, a gift from Alice Ting (Addgene plasmid # 135456; http://n2t.net/addgene:135456; RRID: Addgene_135456) and *Escherichia coli* BL21 (DE3) (ThermoScientific, USA). In brief, the expression vectors were transformed into the *E*. *coli* BL21 strain. The transformed *E*. *coli* cells were grown in SOB medium (RPI Research Product International, Mount Prospect, IL) at 37°C until the OD_600_ reached 0.4–0.6. The TEV expression was then induced by cultivation with 1 mM isopropyl-β-d-thiogalactopyranoside (IPTG) at 30°C for 5 h. The cells were collected following by sonication in binding buffer (100 mM Tris, 500 mM NaCl, 5mM Imidazole, 10% glycerol, pH 7.4) and centrifugation at 10,000 × *g*, 4°C for 30 min. After that, TEV was purified from the supernatants using the Hi-Trap Chelating HP column (Cytiva, Marlborough, MA, USA) under native conditions as described previously. The purity of the protein was assessed by Coomassie blue staining following SDS-PAGE gel electrophoresis. The concentration of TEV was determined using a NanoDrop 8000 spectrophotometer (ThermoScientific, USA). Finally, TEV was stored at -80°C until use.

To cleave off the His fusion tag, affinity purified r*Ixs*S17 (3 μg) and TEV (1 μg) were mixed and incubated at 4°C overnight in cleaving buffer (50 mM Tris-HCl, 0.5 mM EDTA, 1 mM DTT, pH 8.0). The r*Ixs*S17 and TEV ratio was empirically determined in preliminary studies. Subsequently, the reaction mix was dialyzed into Hi-Trap chelating column binding buffer and then purified as described above. Fractions of non-tagged-r*Ixs*S17 were expected to elute into flow through and column wash fractions while both His-tagged TEV and non-cleaved r*Ixs*S17 bound onto the affinity column matrix and were recovered into elution fractions. SDS-PAGE with silver staining and western blotting analysis using the His-tag antibody were used to confirm purification of His-tag free r*Ixs*S17. Quantification was done as described above.

#### r*Ixs*S17 deglycosylation

In a 50 μl reaction, 10 μg of r*Ixs*S17 was deglycosylated using 2 μl of protein deglycosylation mix II (NEB, MA, USA) under denaturing condition following the instructions from the manufacturer. The reaction was incubated at 37°C for 16 h. Deglycosylated r*Ixs*S17 was analyzed by SDS-PAGE and silver staining.

### Profiling inhibitor functions against innate immune system proteases

Inhibitory activity of r*Ixs*S17 against 17 serine proteases related to host responses against tick feeding was determined using substrate hydrolysis assays as described [35,48,93]. The serine proteases and their substrates used in this study are listed in S2 Table. 1μM of r*Ixs*S17 (His-tagged at N- or C-terminal or His-tag removed) was incubated with empirically verified serine protease amount at 37°C for 15 min in reaction buffer (20 mM Tris–HCl, 150 mM NaCl, 0.1% BSA, pH 7.4). Subsequently, 200 nM of the appropriate peptide substrate was added to the final reaction volume of 100 μL and hydrolysis was monitored at 405 nm wavelength every 11s for 30 min at 30°C using microplate reader (Biotek Synergy H1, Winooski, VT, USA). The assay was performed in duplicates of 3 biological replications. Data were subjected to one-phase decay analysis PRISM 9 to determine plateau values as proxies for initial velocity of substrate hydrolysis or residual enzyme activity.

#### Stoichiometry of inhibition (SI)

Preliminary substrate analysis revealed that molar excess of r*Ixs*S17 inhibited pancreatic trypsin, trypsin IV, and blood clotting factor Xa by more than 70%–nearly 100%. To estimate the molar ratio of r*Ixs*S17 to the target protease (pancreatic trypsin, trypsin IV, and factor Xa) required for 100% inhibition of enzyme activity of the target protease, stoichiometry of inhibition (SI) analysis was done as described [35,48,74]. Various molar ratios of His-tagged r*Ixs*S17 to proteases (0, 2.5, 5, 10, 20, 25, 50) were incubated for 15 min at 37°C with constant concentration of bovine trypsin (1.5 nM) or rat trypsin IV (2.0 nM) or factor Xa (13.9 nM). The colorimetric substrate was added; and residual protease activity was determined as described above. SI or the molar ratio of r*Ixs*S17 to protease was determined by plotting the percentage residual protease activity against serpin to protease ratios, fitting data onto the linear regression in PRISM 9, and extrapolation to the ratio which resulted in total loss of protease activity [94].

#### Affinity constant (*k*_*a*_) calculation

The rate of r*Ixs*S17 inhibiting bovine trypsin, trypsin IV and fXa was determined using the discontinuous method [93, 94]. Different concentrations of r*Ixs*S17 (50, 100, 200, 400, 600 and 1000 nM) were incubated with constant amounts of bovine trypsin (14.6 nM), trypsin IV (12.5 nM) or fXa (13.9 nM) for different periods of time (0, 1, 2, 4, 6, 8, 10 and 15 min) at 37°C. The colorimetric substrate was added; and residual protease activity was assayed as described above. The pseudo-first order constant, *k*_obs_, was determined from the slope of a semi-log plot of the residual protease activity against time. The second-rate constant (*k*_*a*_) was determined by the best fit line slope of the *k*_obs_ values that were plotted against r*Ixs*S17 concentration [94].

#### Homology modeling and prediction of basic patches and docking

Secondary structure modeling of *Ixs*S17 was done on ChimeraX molecular modeling server [95]. The mature *Ixs*S17 protein amino acid sequence was pasted into Alphafold comparative modeling software on ChimeraX server, and the best *Ixs*S17 comparative secondary structure model was reported. Putative basic patches were predicted using the electrostatic potential calculator in ChimeraX. Molecular docking study using the ligand molecule heparin (PubChem ID 22833565) with *Ixs*S17 protein was conducted using Autodock Vina and Auto Dock Tools (ADT) v 1.5.4 from the Scripps Research Institute [96, 97]. The ligand was oriented suppositionally to allow flexible rotation and thus explore the most probable binding positions, while the receptor was kept rigid. The grid maps were calculated by Autogrid which represents the center of active site pocket for the ligand. The generated results were visualized by using PyMOL viewer (https://pymol.org/2/).

#### Glycosaminoglycan (GAG) binding and effect on r*Ixs*S17 function

Secondary structure modeling predicted at least one basic patch in *Ixs*S17 comparative modeling structure. To test if the r*Ixs*S17 basic patch is functional, r*Ixs*S17 binding affinity of glycosaminoglycans (GAGs): heparin (Sigma-Aldrich, MO, USA), chondroitin sulphate A (Sigma-Aldrich), heparan sulphate (Galen Laboratory Supplies, North Haven, CT) and dermatan sulphate (Galen Laboratory Supplies) was done as previously described [48]. A GAG-binding microplate (Galen Laboratory Supplies) was coated with 200 μL of GAG at the concentration of 25 μg/mL in binding buffer (100 mM NaCl, 50 mM Na-acetate, 0.2% Tween, pH 7.2) and incubated overnight at room temperature. After washing with binding buffer, the plate was blocked with 250 μL of 1% bovine serum albumin in PBS for 1 h at 37°C. Thereafter, different concentrations of r*Ixs*S17 (0, 1, 2, 5, 10 and 20 μg/mL) in 200μL of blocking buffer was added and incubated for 2 h at 37°C. After the wash, 200 μL of HRP-conjugated anti-histidine antibody (1:5,000 dilution) was added. Following by addition of 200 μL of 1-step Ultra TMB ELISA substrate (Thermo Scientific), 100 μL of hydrochloric acid (1N) was used to stop the reaction; and the OD_450 nm_ was determined using a microplate reader (Biotek Synergy H1).

#### Heparin binding assay

Approximately 300 μg of r*Ixs*S17 or rAAS19 (positive control) or r1E1 (negative control) was applied to the Hi-Trap heparin column (Cytiva). After washing with 10 mM phosphate buffer pH 7.4, the proteins were eluted using a gradient concentration (0.25–0.5–1.0-.2.0–3.0 M) of NaCl. Samples included the protein before binding, flow-through, wash, and elution were collected, resolved on 10% gel of SDS-PAGE, and subjected to silver staining for analysis.

#### Recalcification time assay

Prompted by preliminary findings that r*Ixs*S17 was an inhibitor of blood clotting factors and it bound GAGs including heparin, we assayed the effect of heparin and r*Ixs*S17 mixture on plasma clotting in a recalcification time assay, which evaluates the blood clotting system holistically [98]. Five groups: (1) Buffer control (20 mM Tris-HCl, 150 mM NaCl pH 7.4), (2) 17 kDa heparin sodium salt (0.5 μg/mL) (Sigma-Aldrich, USA), (3) r*Ixs*S17 (2 μM: empirically determined to delay plasma clotting by more than 60 seconds), (4) r*Ixs*S17 and heparin mixture incubated separately, and (5) r*Ixs*S17 and heparin incubated together were incubated in 40 μL of 20 mM Tris-HCl, 150 mM NaCl, pH 7.4 buffer for 5 min at 37°C. Subsequently, 50 μL of pre-warmed (37°C) universal coagulation reference human plasma (UCRP) (ThermoScientific, USA) was added to each group and incubated at 37°C for an additional 5 min. After adding 10 μL CaCl_2_ (final concentration of 150 mM) to trigger plasma clotting, optical density (*A*) was monitored at 650 nm wavelength every 20s for 20 min using the microplate reader (Biotek Synergy H1). Data from the recalcification time assay were plotted onto sigmoid line in PRISM 9. Initiation of plasma clotting (or clotting time) was interpolated from the sigmoid line when *A*_650nm_ increased by 10%, with 95% confident interval as published [74].

#### Differential Precipitation of Proteins (DiffPOP) and *in silico* protein to protein interactions

Differential precipitation of protein-to-protein interaction between r*Ixs*S17 and human plasma was done as described [99]. In a 1.5 mL vial, a reaction mix of 150 μL reaction containing 25 μg r*IxsS17* and 20 μL of human plasma in reaction buffer (20 mM Tris–HCl, 150 mM NaCl, pH 7.4) were incubated at 37°C overnight. Human plasma only and r*IxsS17* only were also incubated in reaction buffer as negative controls. After the incubation, 100 μL of Phosphoprotein Kit- Buffer A (Clontech Laboratories, New York, NY) was added to stabilize the reaction prior to fractionation. To fractionate, precipitation solution (90% methanol/ 1% acetic acid) was added to the stabilized reaction mix, vortexed and incubated at room temperature for 5 min. Precipitates were collected by centrifugation at 14,000 rpm (or max speed) at 4°C. The supernatant was transferred into a new 1.5 mL tube and process repeated until desired fractions are obtained (10 fractions in total). The pellet was washed in 400 μL of ice-cold acetone, air-dried, re-suspended in 100 μL reaction buffer and stored in -80°C until use.

The expectation for this approach is that r*Ixs*S17 will co-precipitate with its interactors. To determine fractions that co-precipitated with r*Ixs*S17, each fraction was resolved on 10% SDS-PAGE gels and subjected to standard western blotting analysis using the monospecific antibody to r*Ixs*S17. The monospecific antibody to r*Ixs*S17 was purified from immune serum of rabbits that were repeatedly fed on by *I*. *scapularis* as previously described [24,48]. The positive signal was developed using chemiluminescent substrates (ThermoScientific, USA).

Fractions that contain potential complexes with r*Ixs*S17 were processed for LC-MS/MS analysis using the method published previously [25]. To identify proteins, extracted tandem mass spectra was searched against the database of non-redundant human proteins from GenBank using the Prolucid program in the Integrated Proteomics Pipeline (IP2) as published [100]. The parameters used to identify potentially true r*Ixs*S17 interactors included detecting at least two peptides in two of three independent LC-MS/MS runs and normalized spectral abundance factors (index for relative protein abundance in exceeded plasma only control values). Subsequently, these interactions were validated using *in silico* methods on the PSOPIA (prediction server of protein-to-protein interactions; https://mizuguchilab.org/PSOPIA/) server [101] and readouts with more than 75% likelihood to interact were considered as true.

#### Complement activity assay

Following up on protein-to-protein interaction results that r*Ixs*S17 also interacted with complement system factors, we investigated its effect on complement pathway activations using the WIESLAB Complement Classical, Alternative and Mannose-binding Lectin (MBL) pathway Kits (Malmö, Sweden). The kits allowed for independent assessment of r*Ixs*S17 on the three complement activation pathways as measured by C5b-C9 or Membrane Attack Complex (MAC) deposition. For initial screening of r*Ixs*S17 possible effect on the complement pathways, we started with high dose of r*Ixs*S17, at 4 μM (20 μg) in 100 μL reactions. Subsequently, inhibition activity assessment of a serially dilute r*Ixs*S17 (0.0078–4 μM) was done. First, r*Ixs*S17 was pre-incubated with positive control (human serum provided with the kit) at 37°C for 30 min. Then, the samples were added to the wells (provided in the kits) along with a blank (diluent only), negative control and positive control, and incubated at 37°C for 60 min. The assay was performed in duplicates. After the washing step, conjugate and substrate were subsequently added following the instructions of the manufacturer. Finally, absorbance was read at 405 nm on a microplate reader (Biotek Synergy H1, Winooski, VT, USA). The effect of r*Ixs*S17 on MAC deposition was calculated as follow: (Sample-NC)/(PC-NC) x100 where NC is negative control and PC is positive control.

#### *Borrelia burgdorferi* complement sensitivity assay

Prompted by preliminary findings that r*Ixs*S17 dose dependently reduced deposition of the MAC, we assessed its effect on rescuing complement sensitive spirochete as previously described [61,62,102]. The complement sensitive *B*. *burgdorferi* (B314/pBBE22*luc*) and complement resistant (B314/pCD100) strains were kindly gifted by the Skare lab (TAMU Health Science Center). Both strains were propagated in BSK-II media at 32°C, 1% CO_2_. For the assay, 15 μL of normal human serum (NHS) (Complement technology, TX, USA) was pre-incubated with serial dilutions of either C-terminal-His/non-tagged r*Ixs*S17 (0.25, 0.5, 0.75, and 1μM), heat-inactivated r*Ixs*S17 (1μM) or protein buffer (PBS; Phosphate buffered saline) at 37°C for 30 min prior to addition of 85 μl of *B*. *burgdorferi* B314/pBBE22*luc* at the concentration of 10^6^ cells/mL and inoculated in a bio-shaker at 32°C, 100 rpm. NHS with *B*. *burgdorferi* B314/pBBE22*luc* or B314/pPCD100 were included as negative and positive controls. Survival of spirochetes was assessed at 1.5, 2, 2.5 and 3 h post incubation. Spirochetes were counted from randomly chosen fields (10–15 fields) under dark-field microscope. Spirochete viability was judged based on cell mobility, membrane integrity, and cell lysis as described [102]. Spirochete survival rates were calculated from 3 biological replicates. Heat-inactivated NHS (hiNHS) was used as the no complement-activity control and for normalization.

#### Effect of *Ixs*S17 on *B*. *burgdorferi* colonization of C3H/HeN Lyme disease mouse model

Routinely, *B*. *burgdorferi* strain B31 (MSK5; kindly gifted by the Skare lab) were cultured in BSK-II medium and virulence plasmid Ip25 and Ip28-1 were verified using PCR primers (S3 Table) as described [103]. Groups of C3H/HeN mice (Charles River Laboratories, Wilmington, MA) (4 mice/group) were intradermally inoculated with 10^4^ *B*. *burgdorferi* spirochetes or 10^4^ *B*. *burgdorferi* spirochetes with various amounts of r*Ixs*S17 (0.06, 0.125, 0.25, 0.5, 1, 2, 5 and 10 μM). Another group of 4 mice were inoculated with BSK-II + PBS as the negative control. At 21 days post inoculation, blood and tissue samples were collected from all mice. Serum was extracted from blood; and genomic DNA was extracted from tissues using DNeasy Blood and Tissue kit (Qiagen, MD, USA). *B*. *burgdorferi* infection was assessed by ELISA, western blot, *in-vitro* cultivation, PCR, and real-time qPCR methods.

#### ELISA

ELISA was used to determine IgM and IgG antibody titer to *B*. *burgdorferi* in mouse sera. Ninety-six-well-microplates (Nunc MaxiSorp, ThermoScientific) were coated with 200 ng/well of *B*. *burgdorferi* lysate antigen, blocked with 5% skim milk at 4°C overnight and incubated with serially diluted mouse sera (at 1:250-500-1,000–2,000) at room temperature for 2 h. Signal was detected using either goat-anti mouse IgM antibody-HRP conjugated (ThermoScientific) or Clean-Blot IP detection reagent (ThermoScientific) at a 1:5,000 dilution, following by addition of the TMB substrate (3,3’,5,5’-tetramethylbenzidine) (ThermoScientific). The reaction was stopped using 2N sulfuric acid and absorbance was read at 450 nm using the microplate reader (Biotek Synergy H1).

#### Western blotting

Two μg of Bb lysate was resolved by SDS-PAGE and transferred to PDVF membrane. The membrane was blocked in 5% skim milk and then incubated with mouse antisera (diluted to 1:200) overnight at 4°C. The anti-mouse IgM-HRP conjugates (ThermoScientific) at 5,000-fold dilution was used to detect primary antibodies. Finally, signal was detected using SuperSignal West Femto Maximum Sensitivity Substrate (ThermoScientific) under Biorad ChemiDoc MP Imaging system.

#### *In-vitro* cultivation of *B*. *burgdorferi*

*In-vitro* cultivation of *B*. *burgdorferi* was used to confirm colonization in mice organs. Within 1 h of completing necropsy, mice organs were submerged in 3–5 mL of BSK-II with appropriate antibiotics and antifungals and incubated at 32°C, 1% CO_2_. The culture was examined bi-weekly for the presence of *B*. *burgdorferi* under dark-field microscope.

#### Real-time quantitative PCR

Real-time qPCR was used to quantify *B*. *burgdorferi* (Bb) in mouse organs targeting Bb *fla*b; and Murine *β-Actin* was used as an internal control and for normalization (Primer sequences are listed in S3 Table) [103,104]. The qPCR assays were performed in 10 μL reactions with 5 μL iTaq Universal SYBR Green Supermix (Bio-rad, Hercules, CA), 300 nM each primer and 10–50 ng mouse organ gDNA on a Bio-rad CFX96 real time system (Bio-rad). Data was analyzed by the comparative (C_t_) method with the equation: Fold change = 2^-ΔΔCt^ = [(C_t_ Flab—C_t_ β-Actin) Bb-r*Ixs*S17 co-injected group—(C_t_ Flab—C_t_ β-Actin) Bb only group] [105]. The spirochete burden in mouse organs was expressed as the fold change of *B*. *burgdorferi* load in mice that were co-injected with r*Ixs*S17 compared with mice injected with *B*. *burgdorferi* only.

### Statistical analysis

Data was analyzed using GraphPad Prism 9 software and represented as mean ± SEM with statistical significance (P < 0.05) detected using the Student’s t-test and two-tailed ANOVA.

## Supporting information

S1 Fig Multiple sequence analysis of *Ixs*S17 and its homologs. Amino acid sequences of *Ixs*S17 and its homologs as well as antithrombin III were aligned in MacVector using T-Coffee specifications. The broken line red box denotes the functional domain reactive center loop. Please note that accession numbers are indicated.
(TIF)

S2 Fig Prediction of signal peptides and subcellular localization for EEC18973.1. Subcellular localization software DeepLoc-2.0 predicted extracellular location for EEC18973.1 (A). SignalP 6.0 software predicted the signal peptide for EEC18973.1 after first 53 amino acid were removed (B). The predicted signal peptides were marked with broken green line.
(TIF)

S3 Fig r*Ixs*S17 under native and deglycosylated forms. (A) Native C-terminal-his and non-tagged-*Ixs*S17 were resolved in clear native PAGE following by silver staining analysis. (B) Silver staining image of C-terminal-his and non-tagged-*Ixs*S17 before and after treatment with deglycosylation enzyme under denaturing condition.
(TIF)

S4 Fig Quantitative real-time PCR analysis of *B*. *burgdorferi* load in mice co-inoculated with 1–10 μM of *rIxs*S17. Four mice/group were inoculated with *B*. *burgdorferi* only (10^4^ spirochetes) with or without different amounts of *rIxs*S17 (1-2-5-10 μM). At 21 days post inoculation, *B*. *burgdorferi* burden in mouse heat, ear, joint and bladder tissues was quantified by real-time qPCR method. The data were presented as fold change of the r*Ixs*S17 treated groups in comparison with Bb group (2 ^-ΔΔCt^ = [(C_t_ Flab—C_t_ β-Actin) Bb-*rIxs*S17 co-injected group—(C_t_ Flab—C_t_ β-Actin) Bb only group]). Red arrows indicate decrease on *B*. *burgdorferi* load. Bb: *B*. *burgdorferi*, Bb + r*Ixs*S17: *B*. *burgdorferi* co-inoculated with *rIxs*S17 groups.
(TIF)

S5 Fig IgG titers against *B*. *burgdorferi* in mice co-inoculated with 1–10 μM of *rIxs*S17. IgG titers against *B*. *burgdorferi* lysate antigen were detected using ELISA. Mouse sera was tested at 1:500 dilution. The data were presented as mean ± SEM; each dot is individual mouse. NC = negative control; Bb = *B*. *burgdorferi* group. Bb = *B*. *burgdorferi*, Bb + r*Ixs*S17 = *B*. *burgdorferi* co-inoculated with *rIxs*S17. Statistical significance was evaluated using Student’s t-test in GraphPad Prism 9 (*:P value ≤ 0.05, ns: no significance). Red arrows indicate decrease on IgG titers.
(TIF)

S6 Fig IgG titers against *B*. *burgdorferi* in mice co-inoculated with 0.06–0.50 μM of *rIxs*S17. IgG titers against *B*. *burgdorferi* lysate antigen were detected using ELISA. Mouse sera was tested at 1:250 and 500 dilutions. The data were presented as mean ± SEM; each dot is individual mouse. NC = negative control; Bb = *B*. *burgdorferi* group. Bb = *B*. *burgdorferi*, Bb + r*Ixs*S17 = *B*. *burgdorferi* co-inoculated with *rIxs*S17.
(TIF)

S1 Table Amino acid residue identity between *Ixs*S17 RCL and other *I*. *scapularis* serpins with coverage above 80%. (DOCX)

S2 Table List of proteases and substrates used in the substrate hydrolysis assay. (DOCX)

S3 Table List of oligonucleotide primers used in the study. (DOCX)

S4 Table Percentage complement activity of the human serum treated with r*Ixs*S17. (DOCX)

S5 Table *B*. *burgdorferi* positive rate of mouse tissues by *in-vitro* cultivation. (DOCX)

S6 Table *B*. *burgdorferi* positive rate of mouse tissues by PCR. (DOCX)

S7 Table Survival rates *B*. *burgdorferi* incubated with different concentrations of r*Ixs*S17 *in-vitro*. (DOCX)

S1 File LC-MS/MS analysis of 10 fractions resulted from differential precipitation of protein assay of *Ixs*S17 with human plasma. (XLSX)
