# Supplementary figures and images for: A tick saliva serpin, IxsS17 inhibits host innate immune system proteases and enhances host colonization by Lyme disease agent
Source: PLoS Pathog. 2024 Feb 23;20(2):e1012032. doi: 10.1371/journal.ppat.1012032 (PMC10917276; doi:10.1371/journal.ppat.1012032)

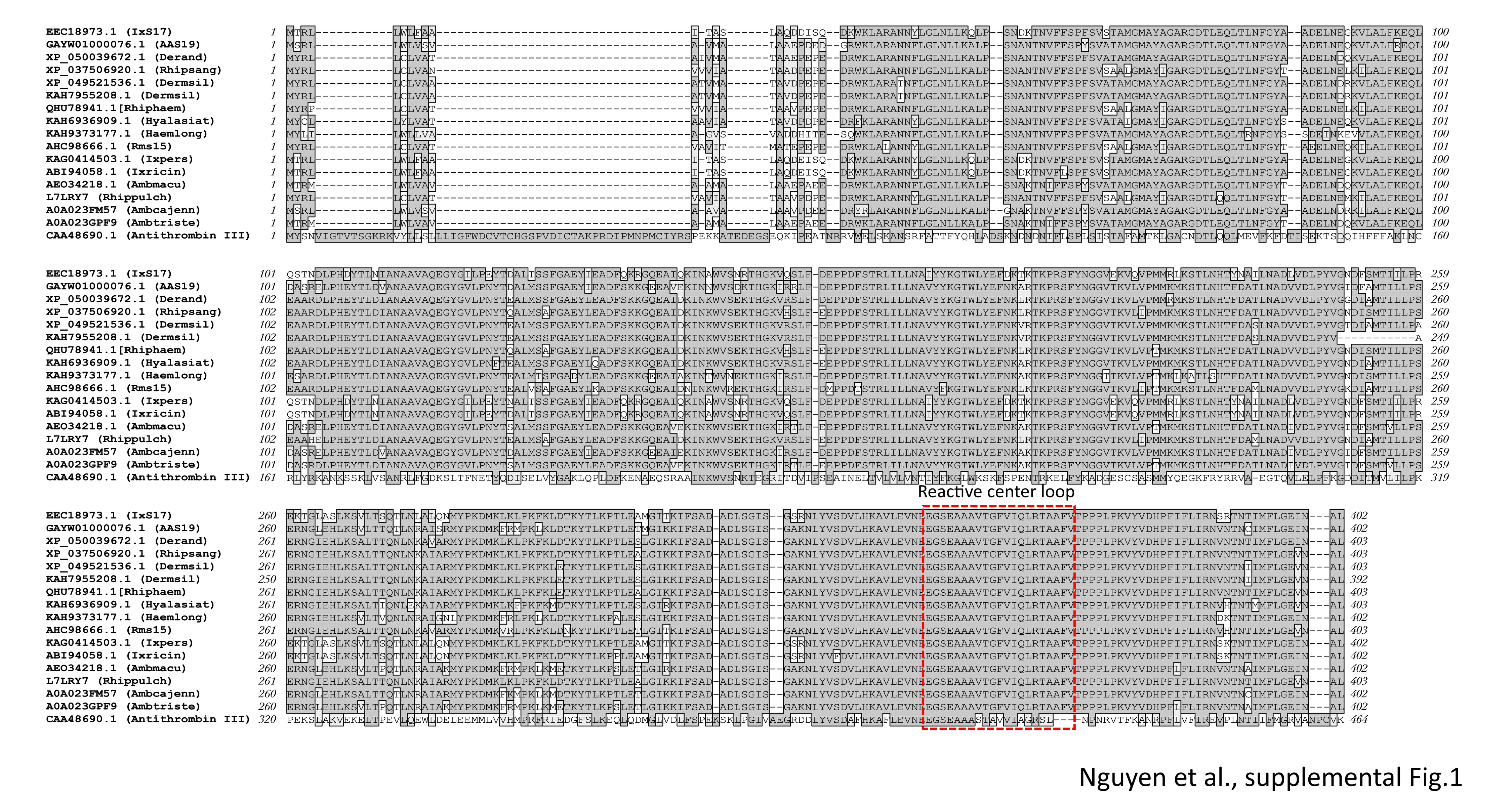

Supplement: S1 Fig — Amino acid sequences of IxsS17 and its homologs as well as antithrombin III were aligned in MacVector using T-Coffee specifications. The broken line red box denotes the functional domain reactive center loop. Please note that accession numbers are indicated. (TIF) [file ppat.1012032.s001.tif]

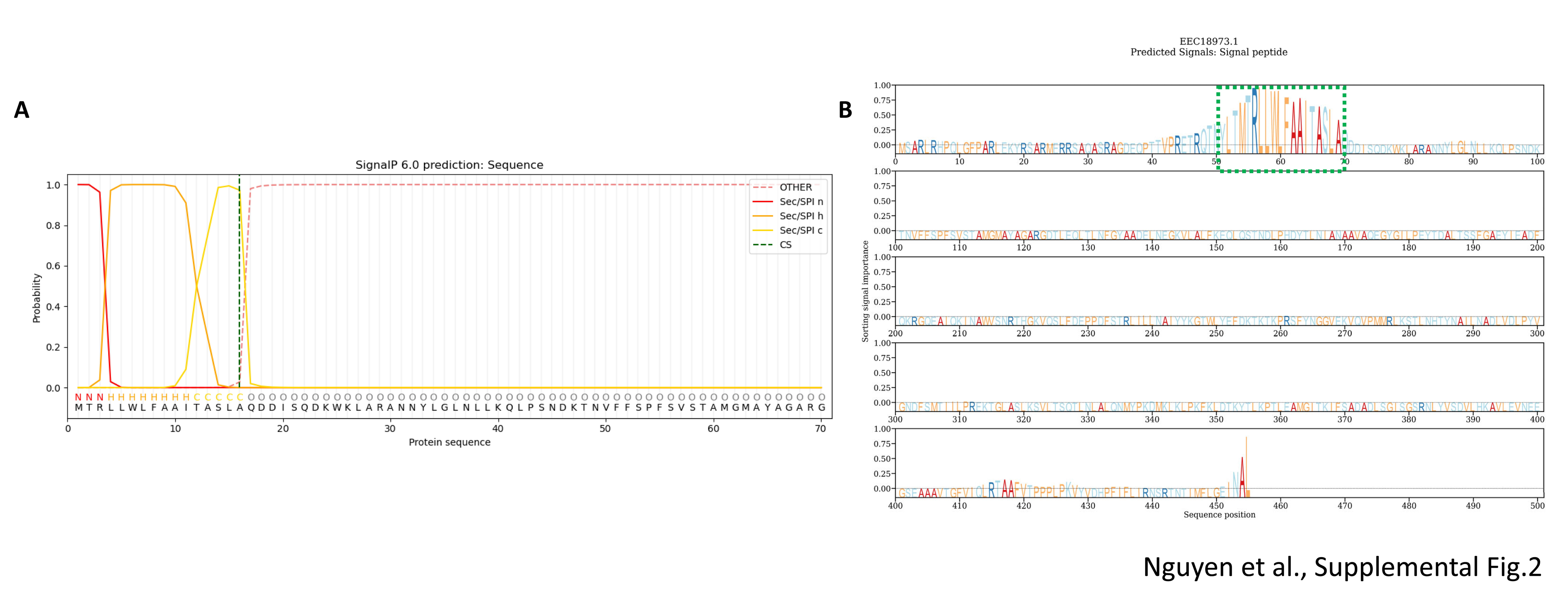

Supplement: S2 Fig — Subcellular localization software DeepLoc-2.0 predicted extracellular location for EEC18973.1 (A). SignalP 6.0 software predicted the signal peptide for EEC18973.1 after first 53 amino acid were removed (B). The predicted signal peptides were marked with broken green line. (TIF) [file ppat.1012032.s002.tif]

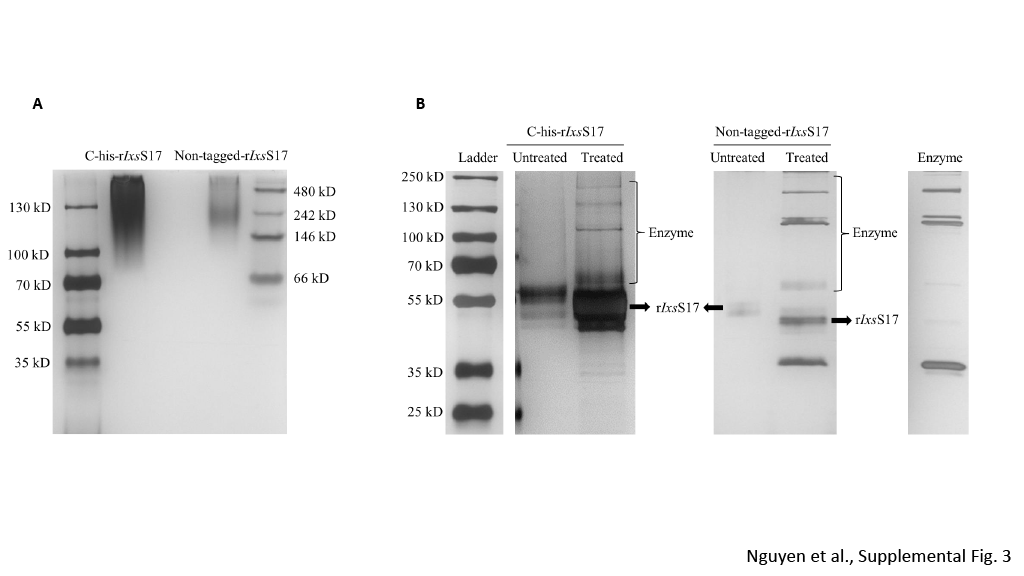

Supplement: S3 Fig — (A) Native C-terminal-his and non-tagged-IxsS17 were resolved in clear native PAGE following by silver staining analysis. (B) Silver staining image of C-terminal-his and non-tagged-IxsS17 before and after treatment with deglycosylation enzyme under denaturing condition. (TIF) [file ppat.1012032.s003.tif]

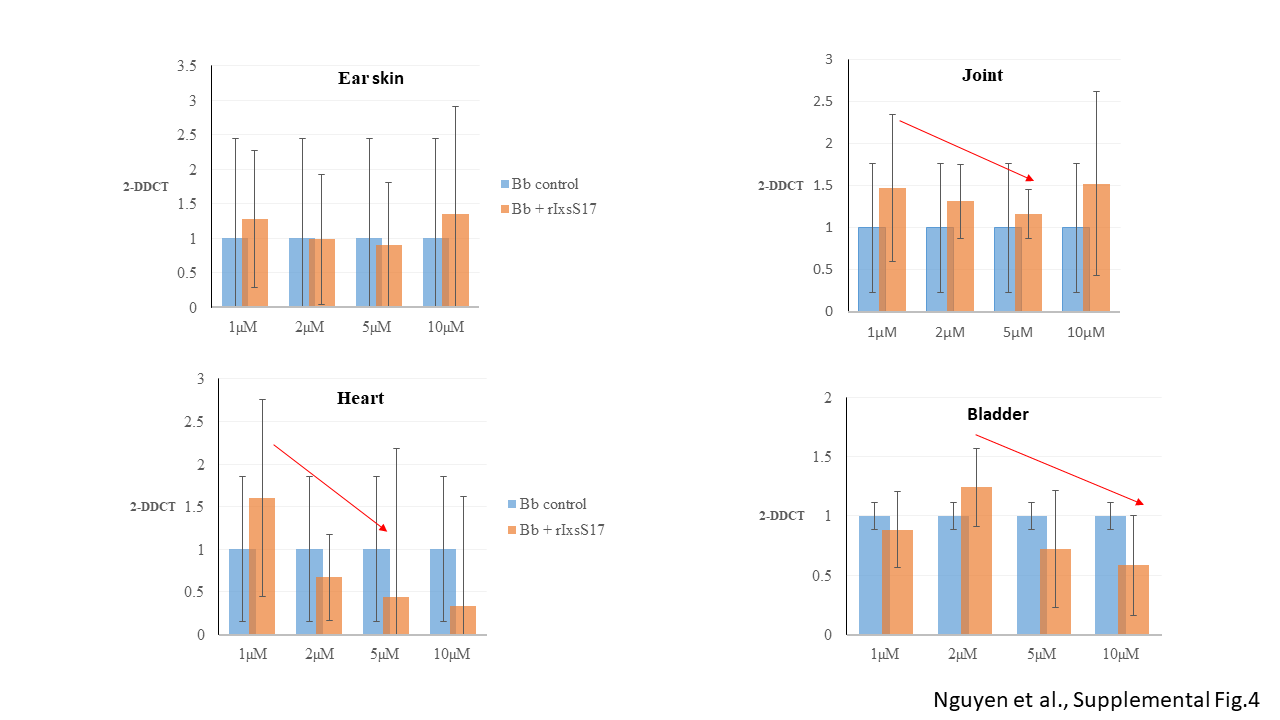

Supplement: S4 Fig — Four mice/group were inoculated with B. burgdorferi only (104 spirochetes) with or without different amounts of rIxsS17 (1-2-5-10 μM). At 21 days post inoculation, B. burgdorferi burden in mouse heat, ear, joint and bladder tissues was quantified by real-time qPCR method. The data were presented as fold change of the rIxsS17 treated groups in comparison with Bb group (2 -ΔΔCt = [(Ct Flab—Ct β-Actin) Bb-rIxsS17 co-injected group—(Ct Flab—Ct β-Actin) Bb only group]). Red arrows indicate decrease on B. burgdorferi load. Bb: B. burgdorferi, Bb + rIxsS17: B. burgdorferi co-inoculated with rIxsS17 groups. (TIF) [file ppat.1012032.s004.tif]

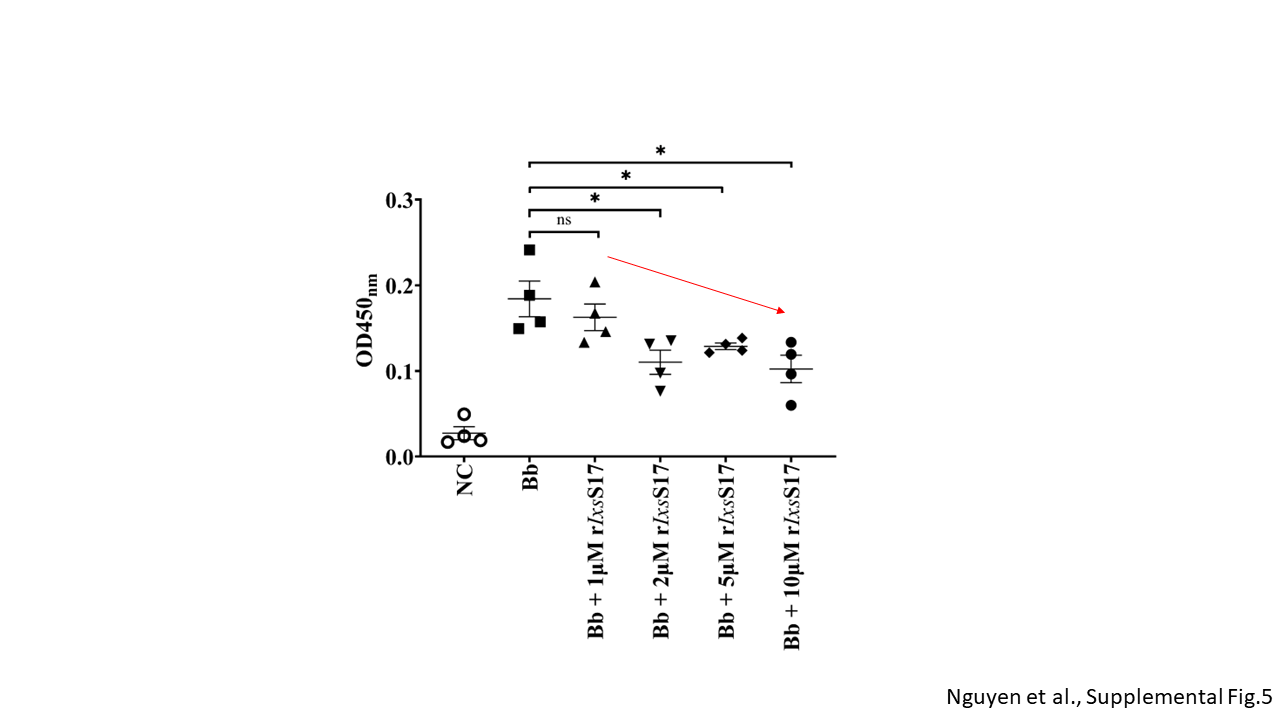

Supplement: S5 Fig — IgG titers against B. burgdorferi lysate antigen were detected using ELISA. Mouse sera was tested at 1:500 dilution. The data were presented as mean ± SEM; each dot is individual mouse. NC = negative control; Bb = B. burgdorferi group. Bb = B. burgdorferi, Bb + rIxsS17 = B. burgdorferi co-inoculated with rIxsS17. Statistical significance was evaluated using Student’s t-test in GraphPad Prism 9 (*:P value ≤ 0.05, ns: no significance). Red arrows indicate decrease on IgG titers. (TIF) [file ppat.1012032.s005.tif]

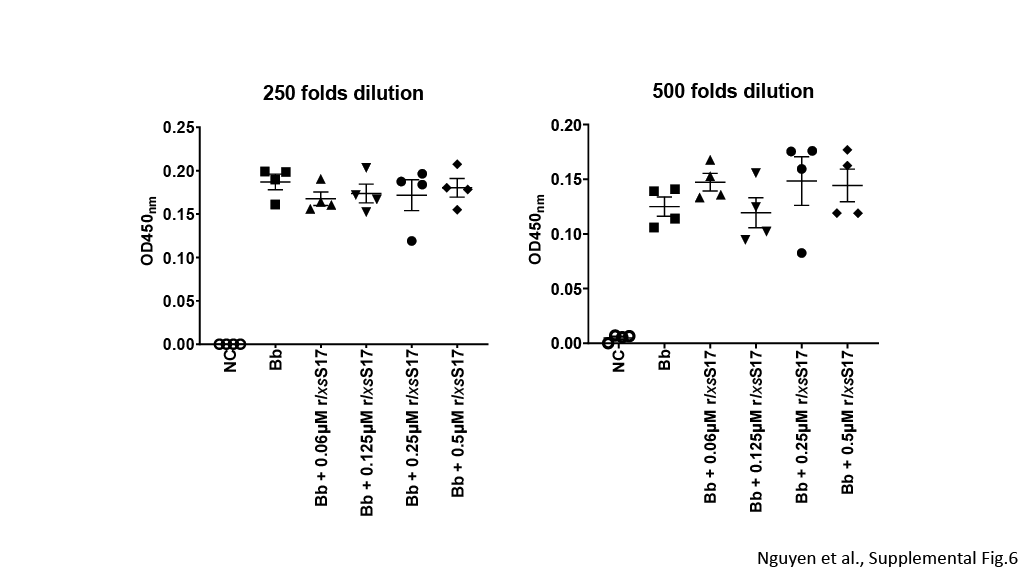

Supplement: S6 Fig — IgG titers against B. burgdorferi lysate antigen were detected using ELISA. Mouse sera was tested at 1:250 and 500 dilutions. The data were presented as mean ± SEM; each dot is individual mouse. NC = negative control; Bb = B. burgdorferi group. Bb = B. burgdorferi, Bb + rIxsS17 = B. burgdorferi co-inoculated with rIxsS17. (TIF) [file ppat.1012032.s006.tif]
