# Supplementary material for: A tick saliva serpin, IxsS17 inhibits host innate immune system proteases and enhances host colonization by Lyme disease agent
Source: PLoS Pathog. 2024 Feb 23;20(2):e1012032. doi: 10.1371/journal.ppat.1012032 (PMC10917276; doi:10.1371/journal.ppat.1012032)
Supplement: S1 Table — (DOCX) [file ppat.1012032.s007.docx]

S1 Table: Amino acid residue identity between *Ixs*S17 and other *I. scapularis* serpin RCLs with coverage above 80%

| **#** | **Accession number** | **Identities (%)** | **Coverage (%)** |
| --- | --- | --- | --- |
| 1 | XP_002415308.5 (*Ixs*S17) | 100 | 100 |
| 2 | XP_002415886.4 | 52 | 85 |
| 3 | XP_029826744.3 | 52 | 95 |
| 4 | XP_029826747.3 | 52 | 95 |
| 5 | XP_040067828.2 | 52 | 89 |
| 6 | XP_042145815.1 | 52 | 86 |
| 7 | XP_029832058.2 | 48 | 100 |
| 8 | XP_042150767.1 | 48 | 84 |
| 9 | XP_040066710.2 | 48 | 90 |
| 10 | XP_040067237.1 | 48 | 95 |
| 11 | XP_029826745.3 | 48 | 95 |
| 12 | XP_002407493.4 | 48 | 95 |
| 13 | XP_002402925.4 | 48 | 90 |
| 14 | XP_040070776.1 | 43 | 100 |
| 15 | XP_029826754.3 | 40 | 100 |
| 16 | XP_029851414.3 | 38 | 90 |
| 17 | XP_029832057.2 | 38 | 83 |
| 18 | XP_029843626.2 | 38 | 95 |
| 19 | XP_042144458.1 | 33 | 94 |
| 20 | XP_040066725.1 | 33 | 90 |
| 21 | XP_042148092.1 | 33 | 86 |
| 22 | XP_002399564.3 | 29 | 81 |
