# Supplementary material for: A tick saliva serpin, IxsS17 inhibits host innate immune system proteases and enhances host colonization by Lyme disease agent
Source: PLoS Pathog. 2024 Feb 23;20(2):e1012032. doi: 10.1371/journal.ppat.1012032 (PMC10917276; doi:10.1371/journal.ppat.1012032)
Supplement: S2 Table — (DOCX) [file ppat.1012032.s008.docx]

|  | **Protease** | **Source** | **Company** | **Molarity (nM)** | **Substrate** | **Company** |
| --- | --- | --- | --- | --- | --- | --- |
| 1 | trypsin IV | rat (recombinant protein) | N/A | 2.0 | N-Benzoyl-L-phenylalanyl-L-valyl-L-arginine-4- nitroanilide hydrochloride | Sigma-Aldrich |
| 2 | trypsin | bovine (pancreas) | Sigma-Aldrich | 1.5 | N-Benzoyl-L-phenylalanyl-L-valyl-L-arginine-4- nitroanilide hydrochloride | Sigma-Aldrich |
| 3 | thrombin | bovine | Sigma-Aldrich | U | N-Benzoyl-L-phenylalanyl-L-valyl-L-arginine-4- nitroanilide hydrochloride | Sigma-Aldrich |
| 4 | elastase | human (neutrophil) | Molecular Innovation | 22.3 | MeOSuc-AAVP | Sigma-Aldrich |
| 5 | elastase | porcine (pancreas) | Sigma-Aldrich | 18.5 | N-Succinyl-tri-L-alanine 4-nitroanilide, N-Succinyl-L- alanyl-L-alanyl-L-alanine 4-nitroanilide | Sigma-Aldrich |
| 6 | chymotrypsin | bovine (pancreas) | Sigma-Aldrich | 1.4 | N-Succinyl-Ala-Ala-Pro-Phe-pNA, N-Succinyl-L-alanyl-L- alanyl-L-prolyl-L-phenylalanine 4-nitroanilide | Sigma-Aldrich |
| 7 | chymase | rat (extract 04/2017) | N/A | U | N-Succinyl-Ala-Ala-Pro-Phe-pNA, N-Succinyl-L-alanyl-L- alanyl-L-prolyl-L-phenylalanine 4-nitroanilide | Sigma-Aldrich |
| 8 | chymase | mice (extract 08/2017) | N/A | U | N-Succinyl-Ala-Ala-Pro-Phe-pNA, N-Succinyl-L-alanyl-L- alanyl-L-prolyl-L-phenylalanine 4-nitroanilide | Sigma-Aldrich |
| 9 | chymase | human (recombinant) | Sigma-Aldrich | 21.7 | N-Succinyl-Ala-Ala-Pro-Phe-pNA, N-Succinyl-L-alanyl-L- alanyl-L-prolyl-L-phenylalanine 4-nitroanilide | Sigma-Aldrich |
| 10 | cathepsin G | human (neutrophil) | Mollecular Innovation | 280.9 | N-Succinyl-Ala-Ala-Pro-Phe-pNA, N-Succinyl-L-alanyl-L- alanyl-L-prolyl-L-phenylalanine 4-nitroanilide | Sigma-Aldrich |
| 11 | factor Xa | bovine | NEB | 2.3 | CS 11 (22) | Hyphen BioMed |
| 12 | factor XIa | human | Enzyme Research Laboratories | 3.7 | CS 31 (02) | Hyphen BioMed |
| 13 | factor XIIa | human | Enzyme Research Laboratories | 15.0 | CS 31 (02) | Hyphen BioMed |
| 14 | kallikrein | porcine (pancreas) | Sigma-Aldrich | 20.0 | CS 31 (02) | Hyphen BioMed |
| 15 | factor IXa beta | bovine | Enzyme Research Laboratories | 314.4 | Penfachrome FIXa | Pentapharm |
| 16 | thrombin | human | Enzyme Research Laboratories | 18.9 | N-Benzoyl-L-phenylalanyl-L-valyl-L-arginine-4- nitroanilide hydrochloride | Sigma-Aldrich |
| 17 | plasmin | human | Enzyme Research Laboratories | 33.7 | S-2251 | Diapharma Group Inc. |

S2 Table. Proteases and substrates used in substrate hydrolysis assay
