# Supplementary material for: A tick saliva serpin, IxsS17 inhibits host innate immune system proteases and enhances host colonization by Lyme disease agent
Source: PLoS Pathog. 2024 Feb 23;20(2):e1012032. doi: 10.1371/journal.ppat.1012032 (PMC10917276; doi:10.1371/journal.ppat.1012032)
Supplement: S3 Table — (DOCX) [file ppat.1012032.s009.docx]

S3 Table: Oligonucleotide primers used in the study

| # | **Primers** | **Sequences** | **References** |
| --- | --- | --- | --- |
| 1 | PCR-Bb Ip25-F | AAGATTGTATTTTGGCAAAAAATTTTC | Labandeira et al., 2001 |
| 2 | PCR-Bb Ip25-R | ATGGGTAAAATATTATTTTTTGGG | Labandeira et al., 2001 |
| 3 | PCR-Bb Ip28-1-F | GTTGCTTTTGCAATATGAATAGG | Labandeira et al., 2001 |
| 4 | PCR-Bb Ip28-1-R | ATGAACAAAAAATTTTCTATTTC | Labandeira et al., 2001 |
| 5 | PCR-Bb FlaB-F | TTCAATCAGGTAACGGCACA |  |
| 6 | PCR-Bb FlaB-R | GGTGCAGCCTGAGCAGTTT |  |
| 7 | qPCR-Bb FlaB-F | TCT TTT CTC TGG TGA GGG AGC T | T.A. Van Laar et al., 2016 |
| 8 | qPCR-Bb FlaB-R | TCC TTC CTG TTG AAC ACC CTC T | T.A. Van Laar et al., 2016 |
| 9 | qPCR-murine β-Actin-F | CAAGTCATCACTATTGGCAACGA | T.A. Van Laar et al., 2016 |
| 10 | qPCR-murine β-Actin-R | CCA AGA AGG AAG GCT GGA AAA | T.A. Van Laar et al., 2016 |

**References:**

1. Labandeira-Rey M, Skare JT. Decreased infectivity in Borrelia burgdorferi strain B31 is associated with loss of linear plasmid 25 or 28-1. Infect Immun. 2001;69(1):446-55. Epub 2000/12/19. doi: 10.1128/iai.69.1.446-455.2001. PubMed PMID: 11119536; PubMed Central PMCID: PMCPMC97902.
2. Van Laar TA, Hole C, Rajasekhar Karna SL, Miller CL, Reddick R, Wormley FL, et al. Statins reduce spirochetal burden and modulate immune responses in the C3H/HeN mouse model of Lyme disease. Microbes Infect. 2016;18(6):430-5. Epub 2016/03/20. doi: 10.1016/j.micinf.2016.03.004. PubMed PMID: 26993029; PubMed Central PMCID: PMCPMC4975942.
