# Supplementary material for: A tick saliva serpin, IxsS17 inhibits host innate immune system proteases and enhances host colonization by Lyme disease agent
Source: PLoS Pathog. 2024 Feb 23;20(2):e1012032. doi: 10.1371/journal.ppat.1012032 (PMC10917276; doi:10.1371/journal.ppat.1012032)
Supplement: S4 Table — (DOCX) [file ppat.1012032.s010.docx]

**S4 Table: Percentage complement activity of the Positive control, Negative control and r*Ixs*S17 treated groups**

| **Groups** | **Classical pathway (%)** | **Alternative pathway (%)** | **MBL pathway (%)** |
| --- | --- | --- | --- |
| Positive control | 100 | 100 | 100 |
| Negative control | 0 | 0 | 0 |
| PC + 4 µM r*Ixs*S17 | 60.6 ± 0.7 | 38.6 ± 1.8 | 1.4 ± 0.2 |
| PC + 2 µM r*Ixs*S17 | NA | NA | 2.9 ± 0.1 |
| PC + 1 µM r*Ixs*S17 | NA | NA | 5.9 ± 0.2 |
| PC + 0.5 µM r*Ixs*S17 | NA | NA | 11.5 ± 0.3 |
| PC + 0.25 µM r*Ixs*S17 | NA | NA | 18 ± 0.3 |
| PC + 0.125 µM r*Ixs*S17 | NA | NA | 17.5 ± 2 |
| PC + 0.0625 µM r*Ixs*S17 | NA | NA | 27.3 ± 0.5 |
| PC + 0.031 µM r*Ixs*S17 | NA | NA | 44.9 ± 2.2 |
| PC + 0.0156 µM r*Ixs*S17 | NA | NA | 60.9 ± 1.5 |
| PC + 0.0078 µM r*Ixs*S17 | NA | NA | 73.1 ± 1.3 |

PC: Positive control, PC + r*Ixs*S17: Positive control sera pre-incubated with various dilutions of r*Ixs*S17. NA: not available.
