# Supplementary material for: A tick saliva serpin, IxsS17 inhibits host innate immune system proteases and enhances host colonization by Lyme disease agent
Source: PLoS Pathog. 2024 Feb 23;20(2):e1012032. doi: 10.1371/journal.ppat.1012032 (PMC10917276; doi:10.1371/journal.ppat.1012032)
Supplement: S6 Table — (DOCX) [file ppat.1012032.s012.docx]

**S6 Table: *B. burgdorferi* positive rate of mouse tissues in PCR**

| **Group** | **Administration** | **Ear skin (n=4)** | **Joint (n=4)** | **Heart (n=4)** | **Bladder (n=4)** | **Total pos animals** |
| --- | --- | --- | --- | --- | --- | --- |
| 1 | PBS + BSKII | 0 | 0 | 0 | 0 | 0 |
| 2 | Bb + PBS | 3/4 | 4/4 | 2/4 | 4/4 | 4 |
| 3 | Bb + 1µM r*Ixs*S17 | 4/4 | 4/4 | 1/4 | 4/4 | 4 |
| 4 | Bb + 2 µM r*Ixs*S17 | 3/4 | 4/4 | 3/4 | 4/4 | 4 |
| 5 | Bb + 5 µM r*Ixs*S17 | 3/4 | 4/4 | 1/4 | 4/4 | 4 |
| 6 | Bb + 10 µM r*Ixs*S17 | 3/4 | 3/4 | 2/4 | 4/4 | 4 |

PBS: Phosphate buffered saline, BSKII: Barbour-Stoenner-Kelly modified medium, Bb: *B. burgdorferi*, Bb + r*Ixs*S17: *B. burgdorferi* co-inoculated with serial dilutions of r*Ixs*S17.
