# Supplementary material for: A tick saliva serpin, IxsS17 inhibits host innate immune system proteases and enhances host colonization by Lyme disease agent
Source: PLoS Pathog. 2024 Feb 23;20(2):e1012032. doi: 10.1371/journal.ppat.1012032 (PMC10917276; doi:10.1371/journal.ppat.1012032)
Supplement: S7 Table — (DOCX) [file ppat.1012032.s013.docx]

**S7 Table: Survival rates of *B. burgdorferi* incubated with different concentrations of r*Ixs*S17 *in-vitro***

| **r*Ixs*S17 concentrations**  **Incubation time** | **0 µM** | **0.06 µM** | **0.125 µM** | **0.25 µM** | **0.5 µM** | **1 µM** | **5 µM** | **10 µM** |
| --- | --- | --- | --- | --- | --- | --- | --- | --- |
| 0.0 h | 98.0 ± 1.1 | 98.0 ± 1.2 | 98.0 ± 1.2 | 98.0 ± 1.2 | 98.0 ± 1.2 | 98.0 ± 1.2 | 98.0 ± 1.2 | 98.0 ± 1.2 |
| 0.5 h | 97.7 ± 2.3 | 97.7 ± 2.3 | 100.0 ± 0.0 | 98.3 ± 1.7 | 98.3 ± 1.7 | 98.3 ± 1.7 | 98.3 ± 1.7 | 96.7 ± 3.3 |
| 3.0 h | 97.7 ± 1.5 | 97.7 ± 1.2 | 96.3 ± 1.9 | 96.3 ± 1.9 | 95.0 ± 2.9 | 98.0 ± 2.0 | 95.7 ± 2.2 | 97.0 ± 1.5 |
| 6.0 h | 96.7 ± 1.7 | 97.0 ± 1.5 | 98.0 ± 2.0 | 96.3 ± 2.0 | 97.3 ± 1.5 | 97.7 ± 1.4 | 98.7 ± 1.3 | 98.0 ± 1.2 |
| 24.0 h | 96.0 ± 2.1 | 96.3 ± 1.8 | 95. 7 ± 2.1 | 97.3 ± 1.3 | 96.7 ± 1.7 | 95.7 ± 2.2 | 96.3 ± 2.0 | 97.0 ± 1.7 |
